# Hypoxia-induced PLOD2 promotes clear cell renal cell carcinoma progression via modulating EGFR-dependent AKT pathway activation

**DOI:** 10.1038/s41419-023-06298-7

**Published:** 2023-11-27

**Authors:** Tao Liu, Wan Xiang, Zhizhuang Chen, Gang Wang, Rui Cao, Fenfang Zhou, Zhe Meng, Yongwen Luo, Liang Chen

**Affiliations:** 1 Department of Urology, Zhongnan Hospital of Wuhan University, Wuhan, China; 2 Department of Biological Repositories, Zhongnan Hospital of Wuhan University, Wuhan, China; 3 Tumor Precision Diagnosis and Treatment Technology and Translational Medicine, Hubei Engineering Research Center, Zhongnan Hospital of Wuhan University, Wuhan, China; 4 Department of Urology, Beijing Friendship Hospital, Capital Medical University, Beijing, China; 5 Human Genetics Resource Preservation Center of Hubei Province, Wuhan, China; 6 Laboratory of Precision Medicine, Zhongnan Hospital of Wuhan University, Wuhan, China

**Keywords:** Renal cell carcinoma, Growth factor signalling

## Abstract

Clear cell renal cell carcinoma (ccRCC) is a type of kidney cancer that is both common and aggressive, with a rising incidence in recent decades. Hypoxia is a key factor that plays a vital role in the tumorigenesis and metastasis of malignancy. However, the precise mechanisms of hypoxia driving ccRCC progression were not totally uncovered. Our study found that hypoxia level was elevated in ccRCC and might be an independent risk factor of prognosis in ccRCC patients. We identified a key protein PLOD2 was induced under hypoxic conditions and strongly associated with poor prognosis in ccRCC patients. When PLOD2 was depleted, the proliferation and migration of ccRCC cells were reduced in vitro and in vivo, while overexpression of PLOD2 had the opposite effect. Mechanically, the study further revealed that PLOD2 was transcriptionally activated by HIF1A, which binds to a specific promoter region of the PLOD2 gene. PLOD2 was also shown to interact with EGFR, leading to the phosphorylation of the receptor. Furthermore, PLOD2 was responsible for binding to the extracellular domain of EGFR, which ultimately activated the AKT signaling pathway, thus promoting the malignant progression of ccRCC. Treatment with the PLOD2 inhibitor Minoxidil significantly suppressed ccRCC progression by inactivating the EGFR/AKT signaling axis. In summary, the findings of this study shed light on the molecular mechanisms behind PLOD2 expression in ccRCC and suggest that it may serve as a potential predictor and therapeutic target for the clinical prognosis and treatment of ccRCC.

## Introduction

The clear cell renal cell carcinoma (ccRCC) arises from the renal epithelium and is characterized by the presence of clear cells with abundant cytoplasm [1]. Approximately 70–80% of renal cell carcinomas are ccRCC, which is the most common subtype of the disease [2]. Globally, kidney cancer causes more than 100,000 deaths each year [3]. Although advances have been made in the diagnosis and treatment of ccRCC, the mortality rate for patients with metastatic disease is less than 10% after five years [4]. Identifying novel therapeutic targets and understanding the molecular mechanisms underlying ccRCC progression is therefore urgently needed.

Existing clinical data indicate that high levels of intratumoral hypoxia and hypoxia-inducible factor 1 alpha (HIF-1α) expression are among the most important factors in predicting tumor progression and metastatic potential in cancer patients, although the underlying mechanisms of this correlation are still unclear [5]. Tumor cell metastasis is a multi-step complex process where tumor cells are driven (partly due to oxygen and nutrient deficiency) to abandon their origin tissue and establish distant colonization [6]. For example, hypoxia has been shown to promote the release of lysyl oxidase (LOX), a HIF-1α target, derived from tumor cells, which reshapes the collagen proteins in the extracellular matrix (ECM) of the distant microenvironment, thereby facilitating the establishment of “pre-metastatic niches,” as demonstrated in mouse models of breast cancer [7]. Collagen, as one of the major components with the highest abundance in the ECM, is highly dysregulated in terms of expression, post-translational modifications, deposition, and degradation in cancer [8]. Procollagen-lysine, 2-oxoglutarate 5-dioxygenase 2 (PLOD2) is a member of the PLOD gene family and plays a crucial role in mediating the formation of stable collagen cross-links [9]. Mutations in the PLOD2 gene can lead to the autosomal recessive inherited disorder known as Bruck syndrome, characterized by osteoporosis, spinal deformities, and joint contractures due to insufficient hydroxylation of collagen type I [10].

Recently, there is growing evidence that PLOD2 plays a critical role in various cancers. It has been reported that in sarcomas and pancreatic cancer, the expression of PLOD2 is regulated by HIF-1α [11, 12], inhibited by microRNA-26a/b in bladder cancer [13], whereas induced by TGF-β in myofibroblasts [14]. In an in situ mouse model of breast cancer, local tumor invasion and lung metastasis were impaired by knocking down PLOD2 in breast cancer cells [15]. PLOD2 promotes breast cancer cell mesenchymal phenotypes and stemness through upregulating succinate [16]. PLOD2 is highly expressed in colorectal cancer tissues and promotes aerobic glycolysis and cell progression by upregulating HK2 [17]. Although the expression of PLOD2 was modulated by multiple factors, the mechanism and function of PLOD2 in ccRCC is still unknown.

Our study demonstrated that PLOD2 is identified as a novel marker for ccRCC progression through, and high PLOD2 expression indicates advanced tumor stage, lymph node metastasis, and poor overall survival. Functional experiments revealed that knockdown of PLOD2 inhibited ccRCC cell proliferation, migration, invasion, and tumor growth in vivo. Moreover, we found that PLOD2 interacts with the extracellular domain of EGFR and regulates the EGFR-dependent AKT pathway by promoting the phosphorylation of EGFR, which subsequently leads to increased cell proliferation and survival. Moreover, HIF1A binds to the PLOD2 promoter and increases its expression under hypoxic conditions. We also demonstrate that targeting PLOD2 enables inhibition of EGFR phosphorylation activation in ccRCC cells, suggesting a potential therapeutic strategy for ccRCC patients. The ccRCC patients may benefit from targeting the PLOD2/EGFR/AKT axis as a prognostic marker and therapeutic target. It may be possible to develop novel strategies for treating ccRCC and improving patient outcomes by conducting further research along this axis.

## Materials and methods

### Dataset collection

TCGA pan-cancer normalized data were downloaded from the UCSC Xena database (https://xena.ucsc.edu/) and GSE65168, GSE59729, GSE40355, and GSE53757 datasets were downloaded from the NCI/NCBI GEO database (https://ncbi.nlm.nih.gov/geo/). The GSE65168 and GSE59729 were used to analyze the hypoxia potential index (HPI) under hypoxia condition. Co-expression network construction and functional prediction analysis were conducted using the GSE40355 dataset. A comparison was made between normal kidney and ccRCC tissues using the TCGA-KIRC, GSE40435, and GSE53757. The R (version 4.1.2) and R Bioconductor packages were used to analyze the data.

### Evaluation of hypoxia level in ccRCC

ssGSEA (single-sample gene set enrichment analysis) is a non-parametric and unsupervised algorithm used to analyze changes in pathway and biological process activity within individual samples of a gene expression dataset. It primarily focuses on gene sets, which are collections of genes that share common biological functions, chromosomal locations, or regulatory patterns [18]. To understand the role of hypoxia in tumorigenesis and investigate the factors associated with hypoxia, the hypoxia potential index (HPI) was modeled based on the enrichment score (ES) of hypoxia regulator genes (HRGs) calculated by ssGSEA [19]. The hypoxia regulator genes were serials of genes upregulated in response to low oxygen levels, obtained from Molecular Signature Database with the term HALLMARK HYPOXIA [20]. In our study, as previously described in our research [21], we employed the ssGSEA algorithm to assess the relative levels of hypoxia potential index (HPI) in ccRCC and normal kidney tissues. On the other hand, potential targets of HIF1A in ccRCC were obtained from Cistrome Cancer which integrated analysis of TCGA molecular profiling data and public transcription factor ChIP-Seq profiles [22].

### Functional and pathway enrichment analyses

The clusterProfiler package in R was used to analyze gene annotation enrichment to determine biological differences between PLOD2 high and low expression groups [23]. Additionally, we also employed the GSVA (gene set variation analysis) package in R to perform ssGSEA analysis and identify biological process differences between different groups [24]. The gene sets of h.all.v2023.1.Hs.symbols were downloaded from the MSigDB of the Broad Institute (https://www.gsea-msigdb.org/gsea/msigdb/index.jsp).

### Weighted gene co-expression network (WGCNA)

WGCNA (Weighted Gene Co-expression Network Analysis) is a systems biology technique based on high-throughput gene expression data [25]. The parameter β is a soft-thresholding power parameter that enhances strong correlations between genes and penalizes weak correlations. Based on this, a hierarchical clustering tree is constructed, where each branch of the tree represents a different gene module. The adjacency matrix is transformed into a topological overlap matrix (TOM), and genes are classified using the TOM method. The correlation between gene expression and hypoxia potential index (HPI) is evaluated using Pearson correlation coefficient. The module with the highest average gene significance is selected as a potential module associated with hypoxia.

### Cell culture and reagents

Cell lines 293T, 786-O, and Caki-1 were provided by the Chinese Academy of Sciences in Shanghai. We maintained the 293T cell line in DMEM, the 786-O cell line in RPMI 1640, and the Caki-1 cell line in McCoy’s 5A. All cell cultures were grown at 37 °C in a humidified incubator containing 5% CO_2_ with 10% fetal bovine serum (FBS) (Gibco, Thermo Fisher Scientific, USA). Antibodies against FLAG (F1804, Sigma), HA (TA180128, OriGene), GAPDH (sc-365062, Santa Cruz), PLOD2 (66342-1-Ig, Proteintech), HIF1A (20960-1-AP, Proteintech), EGFR (ab52894, Abcam), p-EGFR (4407S, CST), AKT (4691L, CST), p-AKT (4060L, CST), mTOR(2983, CST), p-mTOR (ab109268, Abcam), PI3K (4257T, CST), p-PI3K(4228S, CST), GSK3B (12456S, CST), p-GSK3B (5558S, CST), Snail (3879S, CST), E-cad (3195S, CST), N-cad (13116S, CST), Vimentin (5741S, CST), p-P44/42 (CST, cat# 4370), KI67 (ab15580, Abcam), β-Actin(sc-47778, Santa Cruz) and drugs Gefitinib (S1025, Selleck), Minoxidil (M4145, Sigma) were purchased from indicated commercial sources. Gefitinib and minoxidil are both dissolved in dimethyl sulfoxide (DMSO), preparing readily usable stock solutions.

### Plasmid and RNA inference

To construct expression plasmids encoding the full-length sequences of human PLOD2 (NM_182943.3), HIF1A (NM_001243084.2), and EGFR (NM_001346897.2), commercial synthesis was employed to generate the full-length protein-coding sequences of PLOD2, HIF1A, and EGFR, which were subsequently subcloned into the pcDNA3.1 vector (Obio Technology). The full-length EGFR and the corresponding truncated plasmids were subcloned into a pcDNA3.0-Flag vector. To silence the expression of PLOD2, two small interfering RNAs (siRNAs) targeting PLOD2 were used: PLOD2-siRNA1 (sense: 5′-GCCAGAGCUAAGAAUACAUTT-3′, antisense: 5′-AUGUAUUCUUAGCUCUGGCTT-3′) and PLOD2-siRNA2 (sense: 5′-CAUCAUGAUAGCCGUAUAUTT-3′, antisense: 5′-AUAUACGGCUAUCAUGAUGTT-3′), and a negative control siRNA (sense: 5′-UUCUCCGAACGUGUCACGUTT-3′, antisense: 5′-UAUCGUCUGUGCAAUUAGCTT-3′) was also used. Two siRNAs targeting HIF1A were as follows: HIF-1α-siRNA1 (sense: 5′-CACCAAAGUUGAAUCAGAAdTdT-3′, antisense: 5′-UUCUGAUUCAACUUUGGUGdTdT-3′), HIF-1α-siRNAs (sense: 5′-GCUGGAGACAAUCAUAUTT-3′, antisense: 5′-AUAUGAUUGUGUCUCCAGCTT-3′). The lentivirus expressing short hairpin RNA (shRNA) targeting PLOD2 was also constructed and packaged by Tsingke Biotechnology Co., Ltd (Beijing, China). Lipofectamine 3000 (Invitrogen, Carlsbad, CA, USA) was used for cell transfection following the manufacturer’s instructions.

### Quantitative real-time PCR

RNA extraction is typically performed using RNeasy Plus Mini Kits (Qiagen, Germany) as recommended by the manufacturer. We evaluated the extracted RNA using the NanoDrop instrument from Implen (Germany). Toyobo (Japan) ReverTra Ace qPCR RT Kit is then applied to perform complementary DNA synthesis. Finally, we conducted qRT-PCR with a QuantStudio 6 Flex system from Life Technologies. Table S1 includes primer sequences for the experiments. To quantify gene expression levels, we normalize target gene expression levels to GAPDH, which is used as an internal control.

### Cell proliferation assay

Two assays were used to assess cell proliferation: the colony formation assay and the methyl thiazolyl tetrazolium (MTT) assay. We performed a colony formation assay by seeding 800 cells in an appropriate culture medium into a 6-well plate and allowing them to grow for 14 days. After washing with PBS and fixing with 4% paraformaldehyde, the cells were stained with 0.5% crystal violet. We counted the stained cells to determine proliferation activity. Alternatively, for the MTT assay, we seeded 3000 cells into 96-well plates with 6 replicate wells per condition. Five consecutive plates were retrieved each day, and 20 μl of MTT reagent (5 mg/ml, Sigma-Aldrich) was added to each well. Afterward, each well’s absorbance at 490 nanometers was determined after an incubation period of 4 h. Cellular proliferative activity and growth status were determined using this method.

### Cell migration assay

For the transwell assay, we used polycarbonate transwell filters (Corning, USA) as the experimental material and placed them in a 24-well plate containing 0.2 mL of culture medium without FBS. In the upper chamber, we seeded a total of 5 × 10^4^ cells. Subsequently, we conducted a 24-h incubation and fixed the cells with 4% paraformaldehyde, followed by staining with crystal violet. Additionally, we performed a wound healing assay. Transfected cells were cultured in a 6-well plate until they reached 80% confluence. Then, we vertically scratched the cells using a 200 μL pipette tip and washed the scratched area with PBS. Next, we added 2 mL of culture medium to the wells and further incubated the cells for 12 h. By observing and measuring the cells, we obtained the average gap between the wound edges.

### Immunohistochemical (IHC) analysis

In this study, we employed the following protocol for immunohistochemistry (IHC) detection. Firstly, tissue specimens were fixed in 4% formalin and embedded in paraffin. The paraffin-embedded tissues were then sectioned, deparaffinized, and rehydrated for further processing. Next, blocking was performed at room temperature using 10% goat serum to eliminate non-specific background signals. The tissues were incubated overnight at 4 °C with primary antibodies specific to the target proteins of interest. Following primary antibody incubation, appropriate secondary antibodies were used, and peroxidase-conjugated streptavidin treatment was performed before counterstaining with hematoxylin. Finally, the IHC-detected images were scanned and analyzed using a molecular microscope (Olympus BX53). In this study, tissue microarrays (TMAs) and IHC analysis were also utilized to assess protein expression and distribution, which have been widely applied in previous studies [26]. The IHC staining scores were evaluated using the following criteria: 0 indicated negative staining, 1 indicated weak positive staining, 2 indicated moderate positive staining, and 3 indicated strong positive staining. The positivity rate was assessed based on the percentage of cells showing positive staining, with the scoring as follows: 0 indicated negative staining, 1–25% scored 1, 26–50% scored 2, 51–75% scored 3, and 76–100% scored 4. To calculate the total staining score, the staining intensity score was multiplied by the staining positivity rate score. A total score of 6 or higher was considered as high expression, while a score below 6 indicated low expression.

### Tissue microarray of human ccRCC

This study utilized a tissue microarray (TMA) containing 150 tumor samples and 30 adjacent normal tissue samples, which were purchased from OUTDO Biotech (HKidE180Su02; Shanghai, China). To ensure experimental accuracy, staining was performed according to the technical manual provided with the TMA, which contains comprehensive information regarding the TMA pathology. Detailed information about the manual can be found at https://www.superchip.com.cn/.

### Immunofluorescence assay

After washing with PBS, the 786-O and CAKI-1 cells were fixed in 4% PFA at room temperature for 20 min. The cells were then washed three more times with PBS. At 4 °C, the cells were then incubated with the appropriate antibodies overnight. Fluorescein-labeled secondary antibodies were added and incubated for 40 min at 37 °C after three additional PBS washes. DAPI (D8417, Sigma-Aldrich, Germany) was incubated with the cells for 30 min at room temperature before mounting with mounting medium. A laser scanning confocal microscope (LSM880, Zeiss, Germany) was used to analyze the slides.

### Immunoblot assay

Protease inhibitors (Sigma-Aldrich, USA) were used with RIPA lysis buffer (Beyotime, China) to isolate total cellular protein. Using SDS-PAGE gels (ASPEN), equal amounts of protein were separated and transferred to PVDF membranes (Merck Millipore, USA). Primary antibodies were incubated overnight at 4 °C in Tris-buffered saline with Tween-20 (ASPEN) after membranes were blocked with 5% non-fat milk. The secondary antibodies were then incubated at room temperature for two hours. Enhanced chemiluminescence (ASPEN) was used to visualize protein bands, and a Bio-Rad ChemiDoe XRS+ Imaging System (Bio-Rad, USA) was used to visualize immune response bands.

### Co-immunoprecipitation

Co-immunoprecipitation (Co-IP) was performed according to previously described methods [27]. Cells were lysed with lysis buffer provided by Aspen Biological, and approximately 200 μg of total cellular proteins were incubated with the target antibody overnight at 4 °C. Protein A-agarose beads (#1614813, BIO-RAD) were added, and rabbit control IgG (AC005, Abclonal, Wuhan, China) was included in the reaction as a negative control. After washing with lysis buffer, the precipitates were boiled in SDS sample buffer. An immunoblot test was carried out on the supernatant.

### Dual-luciferase assay

The JASPAR matrix models were utilized to predict potential binding sites of HIF1A on the promoter region of the human PLOD2 gene. Using the predicted sequences of the binding sites, full-length and putative fragments of the human PLOD2 promoter region were constructed into the pGL3-basic plasmid, along with a negative control region from ELK Biotechnology. In addition, an overexpression plasmid for pcDNA3.1-HIF1A was also constructed by ELK Biotechnology. All constructed vectors were verified through sequencing. Co-transfection of specific pGL3-basic PLOD2 promoter plasmids and Renilla luciferase reporter plasmid (pRL-TK) or pcDNA3.1-HIF1A expression plasmid was performed in 293T cells. Luciferase activities were measured 48 h after transfection using the Dual Luciferase Reporter Assay System (RG027, Beyotime Biotechnology, Shanghai, China), and the ratio of Firefly/Renilla luciferase activity was calculated.

### Xenograft tumor

In compliance with the National Institutes of Health Animal Use Guidelines, animal experiments were performed under the approval of the Institutional Animal Care and Use Committee of Wuhan University. Male BALB/c-nu mice aged 3 weeks were kept in a specific pathogen-free (SPF) environment and acclimatized for 7 days. The mice were randomly allocated to 2 groups. For the xenograft mice model, Caki-1 cells infected with LV-control (NC group) or LV-shRNA (sh-PLOD2 group) lentivirus vectors (5 × 10^6^ in 100 µl serum-free medium) were subcutaneously injected into the dorsal flank of each mouse. Tumor length and width were measured every 3 days with calipers, and tumor size was calculated using the formula (length × width^2^)/2 in mm^3^. Xenograft growth was monitored for 45 days after injection. At the end of the experiment, all the mice were humanely euthanized to obtain fresh tumors and measure tumor weight. The xenografts were further analyzed through hematoxylin and immunohistochemical staining for the indicated antibodies.

### Statistical analysis

The statistical analysis and figure formatting were performed using R software (version: 4.1.2) and GraphPad Prism version 6.0 (GraphPad Software, USA). The continuous data were reported as mean ± SD. The two-tailed t-test was used to analyze normally distributed data, while non-parametric Kruskal–Wallis test was used for non-normally distributed data. One-way ANOVA was used to compare multiple groups. Spearman’s test was employed to investigate the correlations between variables. Kaplan–Meier method was used to compare the survival rates. Univariate and multivariate Cox proportional-hazard models were applied to identify independent prognostic factors for ccRCC patients. A *p*-value less than 0.05 was considered statistically significant.

## Results

### High hypoxia level indicates worse prognosis in ccRCC

**Fig. 1:**
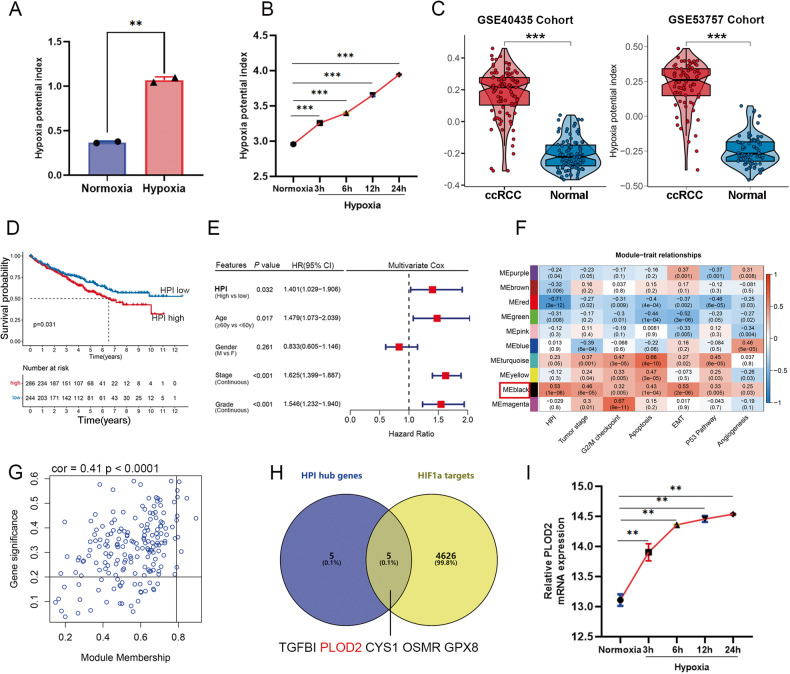
PLOD2 is upregulated under hypoxic condition in ccRCC. **A** Analysis of cohort GSE65168 indicated the hypoxia potential index (HPI) is increase hypoxia condition in ccRCC 786-O cells. **B** Analysis of cohort GSE59729 showed that the HPI increased markedly under hypoxia condition in a time-dependent manner in liver cancer huh7 cells. **C** Analysis of two different cohorts GSE40435 and GSE53757 compared the HPI between ccRCC and normal kidney tissues. **D** Kaplan–Meier analysis showed the overall survival between low and high HPI in TCGA-KIRC. Statistical significance was determined by the log-rank test of Kaplan–Meier analysis. **E** Analysis of TCGA-KIRC indicated that the HPI was the significantly risky variable in the multivariate Cox regression analysis among clinicopathological features (HR = 1.401, *p* = 0.032). **F** Heatmap showing the correlation between genes modules and different biological or clinical characteristics. The red indicated positive correlation and the blue indicated negative correlation. **G** Scatter plot for correlation between gene module membership in the black module (HPI highly correlated) and gene significance. **H** The Venn plot showed the intersection between the hub genes of black module and potential targeted genes of HIF1A. **I** Analysis of cohort GSE59729 showed that the PLOD2 mRNA increased markedly under hypoxia condition in a time-dependent manner. Statistical significance was determined by two-tailed Student’s t test. ***p < 0.001, **p < 0.01.

Differential expression analysis of hypoxia regulator genes (HRGs) was performed between tumors and adjacent normal tissues showed that each of the HRGs was differentially expressed in at least one type of cancer (Figure S1A). Almost all HRGs show diverse regulation patterns in different cancers. That may indicate the expression regulation patterns of hypoxia were tumor-specific. To further investigate the factors or biological processes associated with hypoxia, we calculated the hypoxia potential index (HPI) by integrated analysis of all HRGs by single-sample gene set enrichment analysis (ssGSEA), which may be used to indicate the hypoxia level of each tumor samples. We verified the HPI through two independent GEO gene expression datasets of tumor cell lines that were treated with normal or hypoxia condition at different times. The HPI was calculated for the gene expression datasets [28, 29] in renal carcinoma cells 786-O and liver cancer cells huh7 (Fig. 1A, B). The results showed that hypoxia condition increased the HPI markedly in a time-dependent manner. Thus, the HPI could be used to represent the potential level of hypoxia based on the transcriptome data. It is of interest that HPI is significantly higher in kidney renal clear cell carcinomas (KIRC) than in normal kidneys (Figure S1B). Comparing hypoxia levels between cancer and normal tissues in ccRCC microarray datasets GSE40355 and GSE53757 revealed a significant increase in ccRCC (Fig. 1C). Multivariate Cox regression analysis showed that HPI was a critical risk factor for clinical outcome among various cancer-related markers (*P* = 0.003; Fig. 1E), and high levels of HPI in ccRCC patients were associated with a worse prognosis (*P* < 0.05; Fig. 1D). Our findings suggest that HPI plays a significant role in tumor progression and is a major risk factor for survival, independent of the clinicopathological characteristics of patients with ccRCC.

### PLOD2 is upregulated under hypoxic condition in ccRCC

To identify the key genes regulated by hypoxia in renal carcinoma, we then constructed a co-expression network using transcriptome data from ccRCC and HPI, with a soft threshold power set to “4” to obtain a topological matrix with non-scale characteristics (scale-free R^2^ = 0.85) (Figure S1C, D). Ten gene modules were generated (Figure S1E), and the black module was positively related to HPI (r = 0.53, *p* = 1 × 10^−08^), and tumor stage (r = 0.46, *p* = 6 × 10^−05^) and epithelial-mesenchymal transition (EMT, r = 0.53, *p* = 2 × 10^−06^). The black module had the highest significance among the different modules (Fig. 1F), prompting further analysis. We performed pathway analysis on the genes in the black module and found that the pathway enrichment was mainly in hypoxia and EMT (Figure S1F). Following the analysis, 184 candidate genes were verified as hypoxia-related in ccRCC tissues (Fig. 1G, Table S2). Gene significance and module membership of the genes involved in the black module showed a strong positive correlation (r = 0.41, *P* < 0.001, Fig. 1G). The top 10 HPI hub genes with module member (MM ≥ 0.79) and gene significance (GS ≥ 0.20) were identified from the co-expression network: TGFBI, TIMP1, WDR72, PLOD2, GPAT3, CYS1, C1R, OSMR, PTPN3, and GPX8. Considering that HIF1A is a core transcription factor in hypoxia, we made an intersection between the 10 hub genes and transcriptional gene targets of HIF1A, the we finally got 5 genes (Fig. 1H). We analyzed the expression cohort GSE59729 found that PLOD2 mRNA was increased under hypoxic condition in a time-dependent manner (Fig. 1I).

**Fig. 2:**
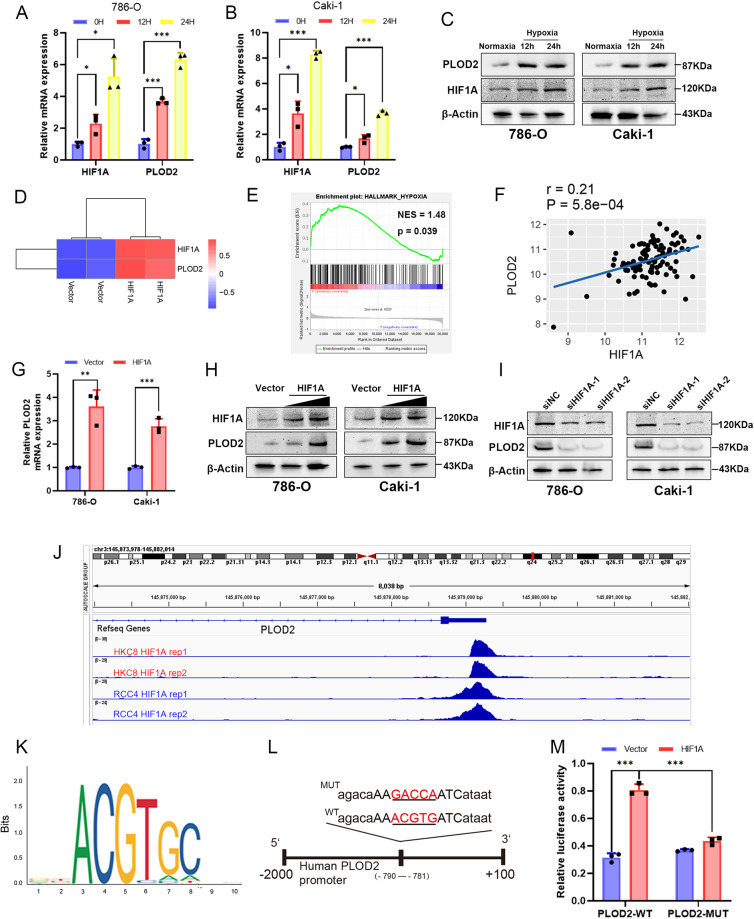
HIF1A increases the expression of PLOD2 gene through transcriptional activation in ccRCC cells. **A**, **B** The expression of PLOD2 and HIF1A mRNA level is increased under hypoxia in 786-O and Caki-1 cells. **C** The protein levels of HIF1A and PLOD2 in ccRCC cells are significantly increased under hypoxia. **D** Analysis of cohort GSE27415 showed the PLOD2 mRNA expression between control and HIF1A overexpression groups in ccRCC 769P cells. **E** GSEA analysis of the cohort GSE53757 indicated PLOD2 is significantly associated with the hypoxia pathway. **F** HIF1A expression is correlated with PLOD2 expression based on cohort GSE73731. **G** HIF1A overexpression promotes PLOD2 mRNA expression. **H** The protein levels of PLOD2 are significantly increased after HIF1A overexpression in ccRCC cells. **I** The protein levels of PLOD2 are significantly downregulated after HIF1A knockdown under hypoxia condition (1% O_2_ for 24 h) in ccRCC cells. **J** GSE120885 ChIP dataset indicates that HIF1A transcriptionally activates PLOD2. **K** The binding site of HIF1A is provided by the JASPAR database. **L** Diagram represents the predicted HIF1A binding site on the PLOD2 promoter, as well as wild-type/mutant PLOD2 promoter plasmids. **M** Luciferase assay in 293T cells co-transfected with PLOD2 luciferase reporter plasmids (pGL4.10-PLOD2 luciferase vector or pGL4.10 base vector), and HIF-1A overexpression plasmid or empty vector. Statistical significance was assessed using two-tailed t tests. Error bars represent the standard deviations of three independent experiments. ***p < 0.001, **p < 0.01, *p < 0.05.

### HIF1A binds to the PLOD2 promoter and increases its expression under hypoxic conditions

The expression of PLOD2 in ccRCC cells was also determined to be induced by hypoxia. PLOD2 and HIF1A expression was enhanced in 786-O and Caki-1 cells when grown in 1% oxygen under hypoxic conditions for 24 h (Fig. 2A–C). In ccRCC 769 P cells, PLOD2 mRNA expression was increased after HIF1A overexpression in cohort GSE27415 (Fig. 2D). Hypoxia plays a major role in the occurrence and progression of ccRCC [30], and GSEA clearly demonstrated that PLOD2 was associated with the hypoxia pathway (Fig. 2E). The transcription factor HIF1A is associated with hypoxic stress and regulates several oncogenes to maintain and promote the growth of blood vessels and tumors in ccRCC [31]. Based on GSE73731 cohort data, we analyzed the association between PLOD2 and HIF1A and found that there was a significant positive correlation between PLOD2 and HIF1A (Fig. 2F). Following this, we investigated whether HIF1A could regulate PLOD2 expression in ccRCC cells under hypoxia. With HIF1A overexpression, PLOD2 expression was found to increase (Fig. 2G, H). Furthermore, we used specific siRNAs to knock down HIF1A under hypoxia conditions, which resulted in decreased expression of PLOD2 (Fig. 2I). As shown in Fig. 2J of the GSE120885 ChIP-seq dataset, HIF1A was transcriptionally activating PLOD2 under hypoxia conditions. To predict HIF1A binding sites on the PLOD2 promoter region, we used the JASPAR database (https://jaspar.genereg.net/). A high-scoring motif was observed between -790 and -781 upstream of the transcription start site, based on the canonical binding motifs of HIF1A (Fig. 2K). We then constructed plasmids containing the wild-type (WT) and mutant (MUT) PLOD2 promoters coupled to HIF1A (Fig. 2L). When HIF1A binding site was mutated, PLOD2-WT promoter-driven luciferase activity was significantly attenuated, while HIF1A expression significantly increased PLOD2-WT promoter-driven luciferase activity (Fig. 2M). Based on the results of this study, it appears that HIF1A binds to the promoter of PLOD2 to activate transcription.

### PLOD2 is highly expressed and could be a prognostic biomarker in ccRCC patients

**Fig. 3:**
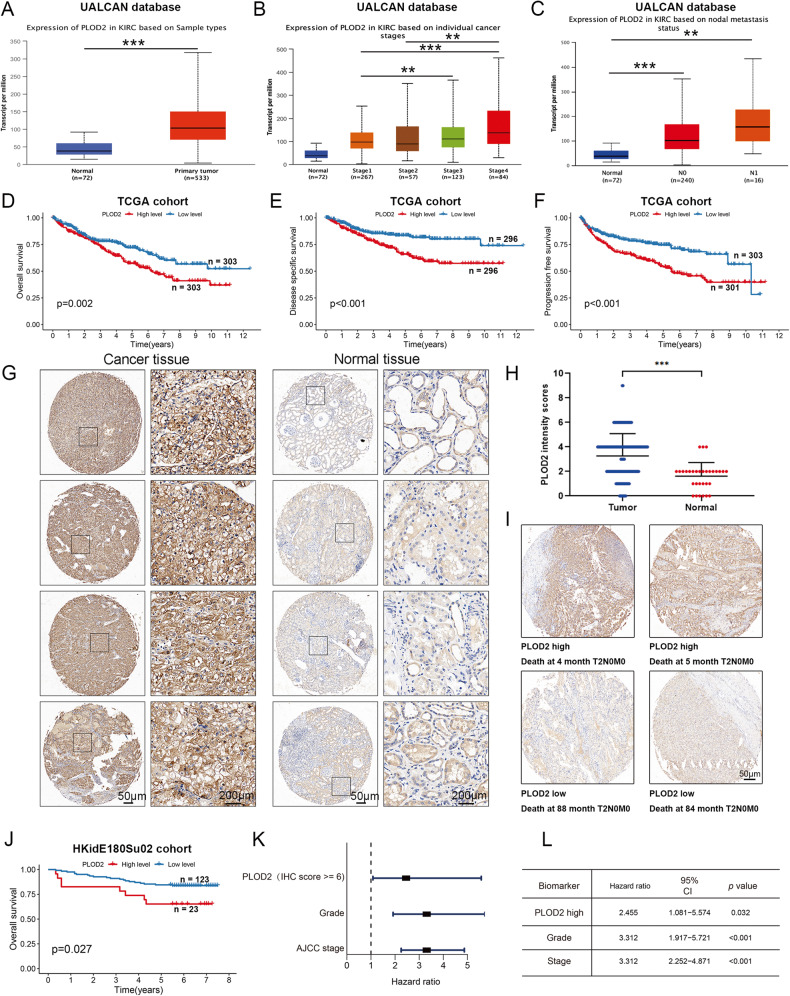
High PLOD2 expression is associated with poor prognosis in patients with ccRCC. **A** Differential expression of PLOD2 gene in ccRCC and normal kidney tissues. **B** PLOD2 expression is associated with higher tumor stage in ccRCC. **C** PLOD2 expression is associated with lymphatic metastasis in ccRCC. **A**–**C** Complete data are obtained from the UALCAN database. **D**–**F** Kaplan–Meier survival analysis of ccRCC patients from the TCGA datasets. Statistical significance was determined by the log-rank test of Kaplan–Meier analysis. **G** Representative PLOD2 IHC images of paired ccRCC tissue and adjacent tissues. The levels of PLOD2 in a ccRCC microarray from the Shanghai OUTDO Biotech (HKidE180Su02) were determined by immunohistochemical staining with an anti-PLOD2 antibody, as described in the “Methods”. Scale bar: 50 μm (second and fourth column); 200 μm (first and third column). **H** PLOD2 is highly expressed in ccRCC tissues compared with normal kidney tissue. The PLOD2 staining intensity was scored in 150 cancer tissues and 30 normal tissues, as described in the “Methods”. **I** Representative images showing high and low PLOD2 staining in the tissue microarray and the survival data of the corresponding patients. The patients included in the microarray were stratified by dichotomizing the PLOD2 expression status in cancer tissues on a continuous score scale of 0–12, with a cut point of 6. **J** Patients with ccRCC with high PLOD2 expression have significantly reduced overall survival. With a cut-off point of median score, Kaplan–Meier survival analysis was performed. **K**, **L** Patients with ccRCC with high PLOD2 expression (PLOD2 high) have significantly increased hazard ratios for death. Patients with ccRCC with low PLOD2 expression were used as the reference to calculate the hazard ratios for death. Statistical significance was assessed using two-tailed t tests. ***p < 0.001, **p < 0.01, *p < 0.05.

We analyzed UALCAN data to evaluate PLOD2 expression in ccRCC clinical samples. As shown in Fig. 3A, PLOD2 was dramatically overexpressed in ccRCC tissues compared to normal kidney tissues. In addition, the expression of PLOD2 was significantly higher in patients with lymph node metastasis as compared to those without lymph node metastasis (Fig. 3C) and increased with increasing tumor stage (Fig. 3B). The Kaplan–Meier analysis of the TCGA-KIRC cohort revealed that higher levels of PLOD2 were associated with a shorter overall survival (OS) time (Fig. 3D). Results from the TCGA-KIRC cohort also revealed that ccRCC patients with high PLOD2 expression had higher disease-specific survival rates (DSS) and progression-free survival rates (DFS) than those with low PLOD2 expression (Fig. 3E, F). As a means of validating the above results, we used ccRCC tissue microarrays (TMA) to measure the expression of PLOD2. A higher level of PLOD2 protein expression was also observed in the tumor tissues of patients with ccRCC compared to normal kidney tissues (Fig. 3G, H). High stage ccRCC expressed more PLOD2 than low stage ccRCC (Figure S2A–F). Patients with cancer with high PLOD2 expression (Fig. 3I, top) were significantly less likely to survive than those with cancer with low PLOD2 expression (Fig. 3I, bottom and Fig. 3J). Furthermore, the multivariate COX proportional hazard analysis of ccRCC patients with high PLOD2 expression indicated a poorer overall survival rate than those with low PLOD2 expression (HR = 2.455, *p* = 0.032, Fig. 3K, L). Thus, these results suggest that PLOD2 may be an important prognostic factor for patients with ccRCC.

### PLOD2 promotes the proliferation and migration capacity of ccRCC cells

**Fig. 4:**
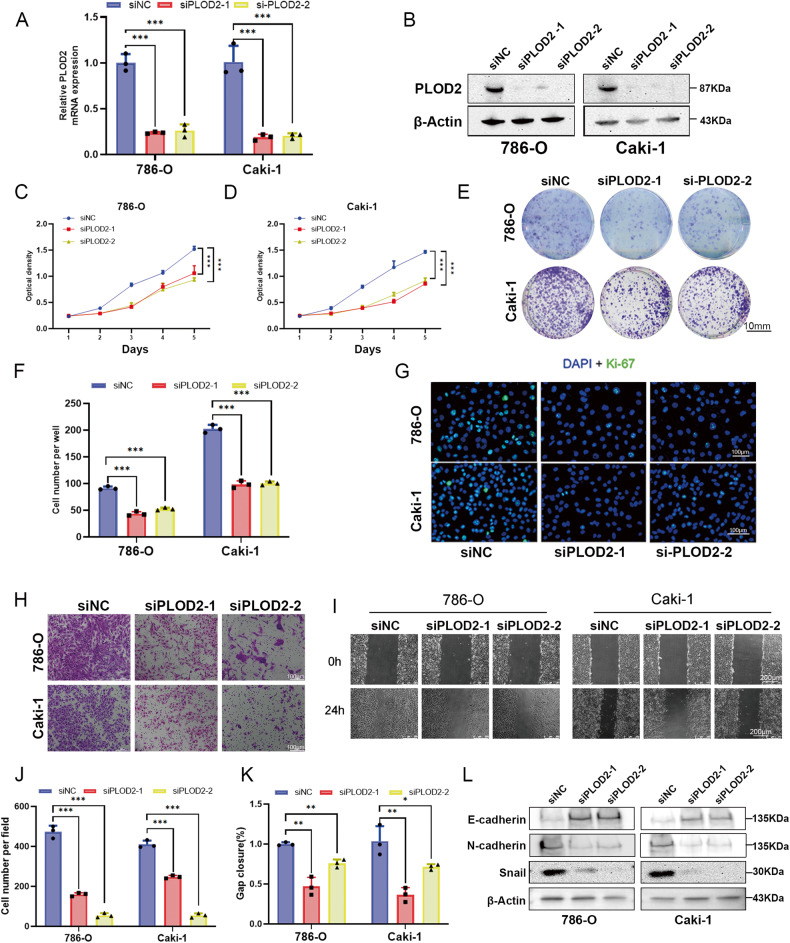
PLOD2 depletion attenuates ccRCC cell proliferation and migration in vitro. **A** The qRT-PCR analysis evaluated the depletion efficiency of two PLOD2-specific siRNA in 786-O and CAKI-1 cells, si-1 and si-2 represent favorable depletion efficiency. **B** Immunoblot analysis validated the depletion efficiency of si-1 and si-2 in 786-O and CAKI-1 cells. **C**, **D** MTT assays revealed that PLOD2 depletion attenuated cell viability in 786-O and CAKI-1 cells. **E**, **F** Clone formation assay and the statistical chart represent PLOD2 depletion attenuated clone formation viability in 786-O and CAKI-1 cells. **G** Immunofluorescence assays examine the effect of PLOD2 deficiency on Ki-67 expression in 786-O and CAKI-1 cells. The scale bar for immunofluorescent staining images is 100 μm. **H**, **I** Transwell assay and wound healing assay demonstrated that PLOD2 depletion inhibits cell migration in 786-O and CAKI-1 cells. **J** The wound healing assay and the statistical chart show that PLOD2 depletion inhibit cell migration in 786-O and CAKI-1 cells. **J**, **K** Statistical charts of transwell assay and wound healing assay. **L** Immunoblot assay shows EMT-related proteins in 786-O and CAKI-1 cells after PLOD2 depletion. Statistical significance was assessed using two-tailed t-tests. Error bars represent the standard deviations of three independent experiments. ***p < 0.001, **p < 0.01, *p < 0.05.

To examine the role of PLOD2 in regulating the proliferation viability of ccRCC cells, we depleted the PLOD2 gene in 786-O and CAKI-1 cells (Fig. 4A, B). As shown in Fig. 4C, D, PLOD2 depletion greatly reduced the viability of cell proliferation (Fig. 4C, D). In addition, PLOD2 depletion significantly decreased clone formation capability (Fig. 4E, F). Ki-67-positive ccRCC cells were significantly reduced when PLOD2 was knocked down using immunofluorescence methods (Fig. 4G). Transwell and wound healing assays were also used to detect the altered migration capacity of 786-O and CAKI-1 cells. PLOD2 depletion reduced the migration capacity of ccRCC cells (Fig. 4H–K). As epithelial-mesenchymal transition (EMT) is considered one of the most common pathological processes leading to tumor progression, EMT markers (E-cadherin, N-cadherin, and snail) were detected via immunoblot analysis. Depletion of PLOD2 increased E-cadherin levels, while N-cadherin and snail levels decreased (Fig. 4L). This suggests that PLOD2 may promote ccRCC cell migration by regulating EMT.

### Inhibition of PLOD2 blocks tumor growth in vivo

**Fig. 5:**
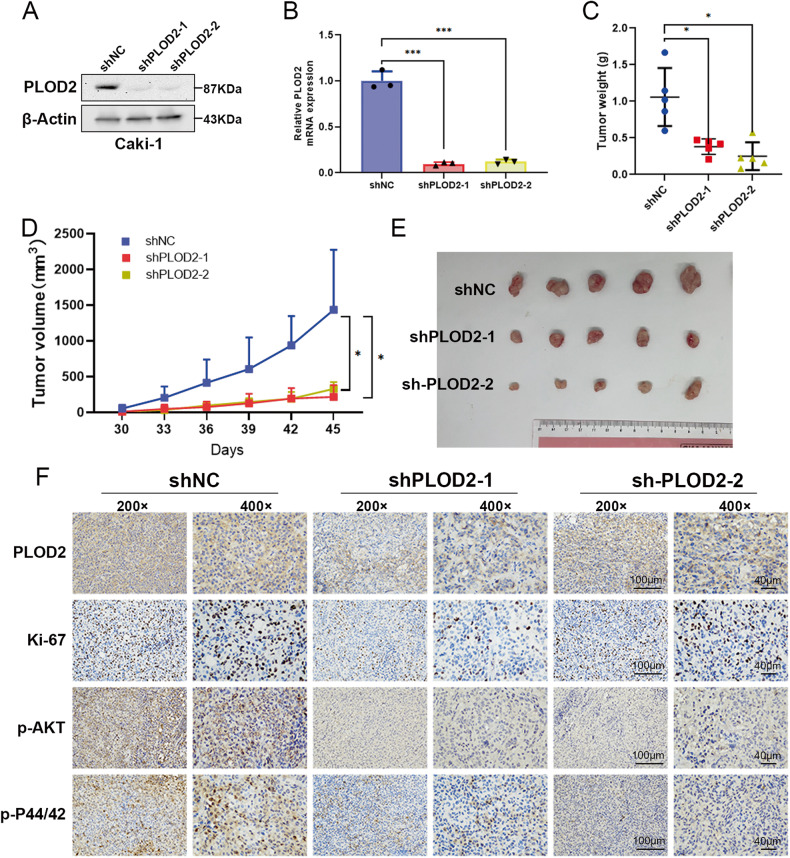
PLOD2 promotes ccRCC cell growth in vivo. **A**, **B** Immunoblot assay and qRT-PCR verified PLOD2 depletion efficiency in CAKI-1 stable cells. **C** PLOD2 depletion significantly decreased the weight of the tumor xenograft (*n* = 5 in each group). **D** PLOD2 depletion significantly inhibited tumor growth (*n* = 5 in each group). **E** Tumor image of xenograft mice models. **F** IHC staining detected the expression of Ki67, PLOD2, p-AKT, and p-P44/42. Data are shown as the mean ± SD. Statistical significance was assessed using a two-tailed t-test. ***p < 0.001, **p < 0.01, *p < 0.05.

Further investigation was performed in the xenograft mouse model to determine whether PLOD2 contributes to tumor growth. By using lentivirus-based shRNA to deplete PLOD2 in Caki-1 cells, the stable depletion efficiency of PLOD2 was detected using qRT-PCR and immunoblot assays (Fig. 5A, B). Xenograft mice models were created by separately injecting Caki-1 LV-control cells (NC) and Caki-1 LV-shPLOD2 cells into BALB/c nude mice. Figure 5C shows that the weight of the tumors in the shPLOD2 group was significantly lighter than that of the NC group. There was a slower tumor growth in the shPLOD2 group than in the NC group (Fig. 5D, E). Moreover, IHC staining revealed that the shPLOD2 group showed lower positive Ki67, p-AKT, p-P44/42 staining than the NC group (Fig. 5F). Therefore, the results indicate that depleting PLOD2 in vivo reduces the growth rate of ccRCC cells.

### PLOD2 interacts with the EGF receptor (EGFR) and modulates its activation

**Fig. 6:**
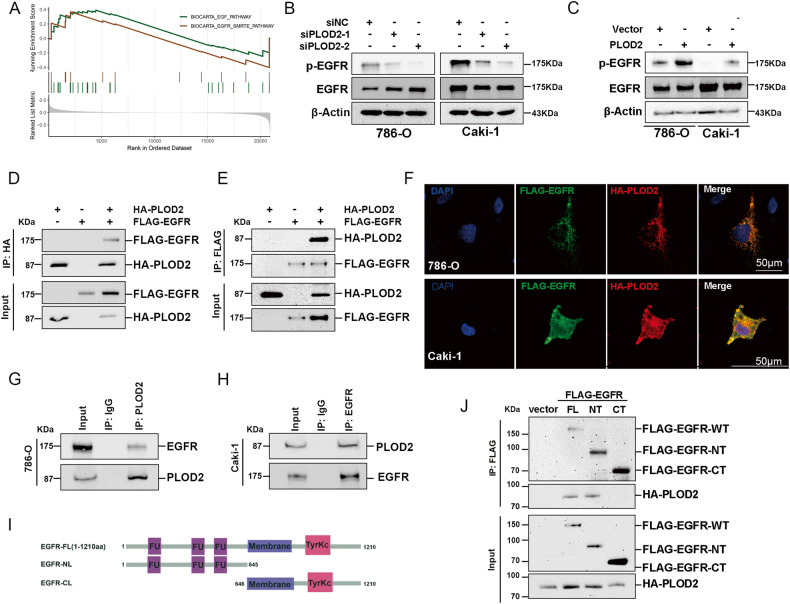
PLOD2 interacts with extracellular domain of EGFR and induces EGFR phosphorylation. **A** GSEA of GSE40435 cohort shows PLOD2 is positively related to the EGFR pathway. **B**, **C** Immunoblot assay elucidates that PLOD2 depletion attenuates EGFR phosphorylation level, while ectopic overexpression of PLOD2 promoted EGFR phosphorylation level. **D**, **E** Exogenous Co-IP assay represents that PLOD2 could interact with EGFR in 293T cells. **F** PLOD2 (green) and EGFR (red) co-localization were examined by immunofluorescence assay (scale bars: 15 μm), the nucleus was indicated by DAPI (blue) staining. **G**, **H** Endogenous Co-IP assay proved PLOD2 could interact with EGFR in 786-O and CAKI-1 cells. **I** Diagram of EGFR-FL, EGFR-NT, and EGFR-CT. **J** Co-IP assay between PLOD2 and EGFR-FL, EGFR-NT, and EGFR-CT in 293-T cell line.

To investigate the potential mechanism of PLOD2 in ccRCC malignant progression, GSEA analysis was conducted based on the TCGA-KIRC dataset. In samples with highly expressed PLOD2, EGFR-related signaling was enriched (Fig. 6A). Depletion of PLOD2 led to an attenuation of the phosphorylated EGFR protein, while total EGFR was unaffected (Fig. 6B). Conversely, ectopic overexpression of PLOD2 increased phosphorylated EGFR protein levels (Fig. 6C). It is speculated that PLOD2 may modulate EGFR phosphorylation by interacting with it. The exogenous Co-IP assay revealed that PLOD2 could interact with EGFR in 293T cells (Fig. 6D, E). In both 786-O and CAKI-1 cells, PLOD2 was co-localized with EGFR by immunofluorescence assay (Fig. 6F). The endogenous Co-IP assay showed that PLOD2 could interact with EGFR in both 786-O and Caki-1 cells (Fig. 6G, H). As a further means of identifying the region of EGFR responsible for its interaction with PLOD2, we separated EGFR into the extracellular and intracellular domains (Fig. 6I), and the results examined how PLOD2 interacted with the extracellular domain of EGFR (Fig. 6J). Overall, these findings demonstrate that PLOD2 interacts with EGFR through its transmembrane regions.

### PLOD2 facilitates the oncogenic functions via the EGFR/AKT signaling axis

**Fig. 7:**
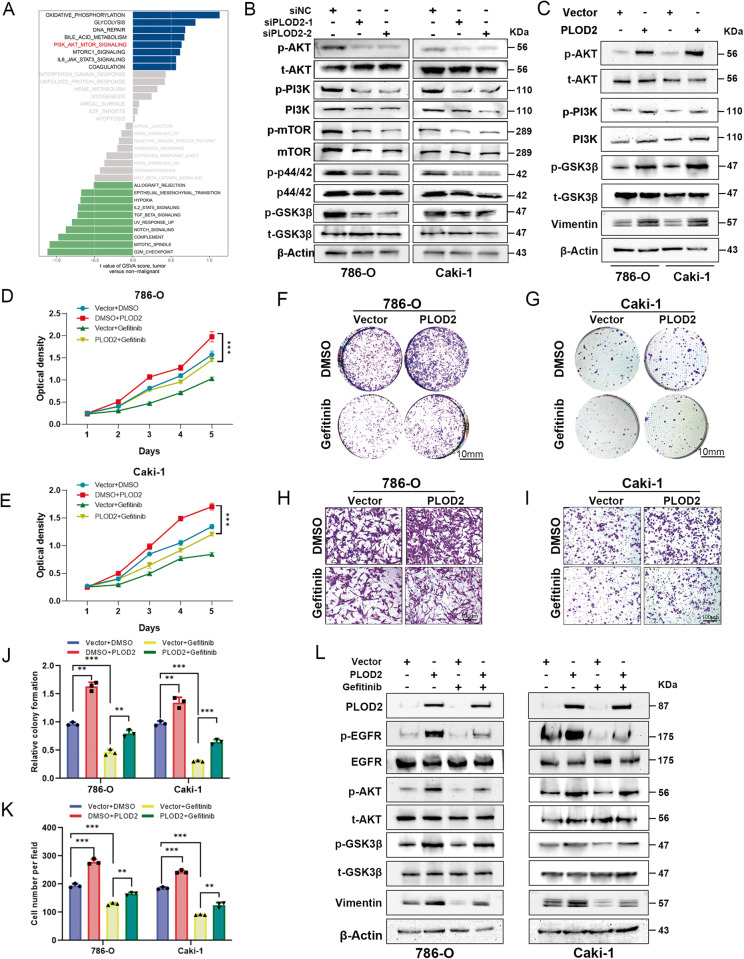
PLOD2 promotes ccRCC cell proliferation through the EGFR/AKT/mTOR pathways. **A** GSVA result indicated AKT pathway was significantly downregulated in the PLOD2-depletion group compared to the control group based on RNA-Seq data of PLOD2-depleted CAKI-1 cells and control cells. **B**, **C** Immunoblot assay elucidates that PLOD2 depletion attenuates AKT, GSK3β, and mTOR phosphorylation levels, while ectopic overexpression of PLOD2 increases AKT and GSK3β phosphorylation levels. **D**, **E** MTT assay elucidates gefitinib attenuated proliferation viability induced by PLOD2 overexpression. **F**, **G** The clone formation assay represents EGFR inhibitor gefitinib attenuated clone formation viability induced by PLOD2 overexpression in 786-O and CAKI-1 cells. **H**, **I** Transwell assay represents gefitinib attenuated migration viability induced by PLOD2 overexpression. **J** Statistical charts of clone formation assay. **K** Statistical charts of transwell assay. **L** Immunoblot results elucidate that gefitinib attenuate p-AKT, p-GSK3β, and Vimentin level induced by PLOD2 overexpression. Statistical significance was assessed using a two-tailed t test. Error bars represent the standard deviations of three independent experiments. ***p < 0.001, **p < 0.01, *p < 0.05.

Our RNA-Seq analysis of PLOD2-depleted CAKI-1 cells and control cells examined downstream signaling pathways and functional processes that were affected by transcriptional regulation. According to the GSVA analysis, the AKT pathway was significantly downregulated in CAKI-1 cells that were depleted of PLOD2 in comparison to controls (Fig. 7A). Following overexpression or knockdown of PLOD2, we detected key proteins involved in AKT pathways. The phosphorylation of AKT, GSK3β and PI3K were downregulated in PLOD2-depleted ccRCC cell lines (Fig. 7B). In contrast, ectopic overexpression of PLOD2 increased the phosphorylation of AKT, GSK3β and PI3K in ccRCC cells (Fig. 7C). Since previous research reports that the AKT pathway is downstream of EGFR, we hypothesized that PLOD2 might play an oncogenic role in ccRCC via the EGFR/AKT signaling axis. To investigate the role of EGFR in PLOD2-induced ccRCC progression, Gefitinib, a specific inhibitor of EGFR, was administered to PLOD2-overexpressed ccRCC cells to monitor the changes in cellular proliferation and migration. It was found that cells overexpressing PLOD2 treated with gefitinib showed reduced proliferation, migration, and clone-formation abilities (Fig. 7D–K). As shown in Fig. 7L, the immunoblot assay indicated that ccRCC cells treated with gefitinib showed an inhibition of the EGFR/AKT signaling axis induced by PLOD2. Overall, these data suggest that PLOD2 promotes ccRCC progression through modulation of the EGFR/AKT signaling pathway.

### Minoxidil inhibits ccRCC progression by inactivating EGFR/AKT signaling axis

**Fig. 8:**
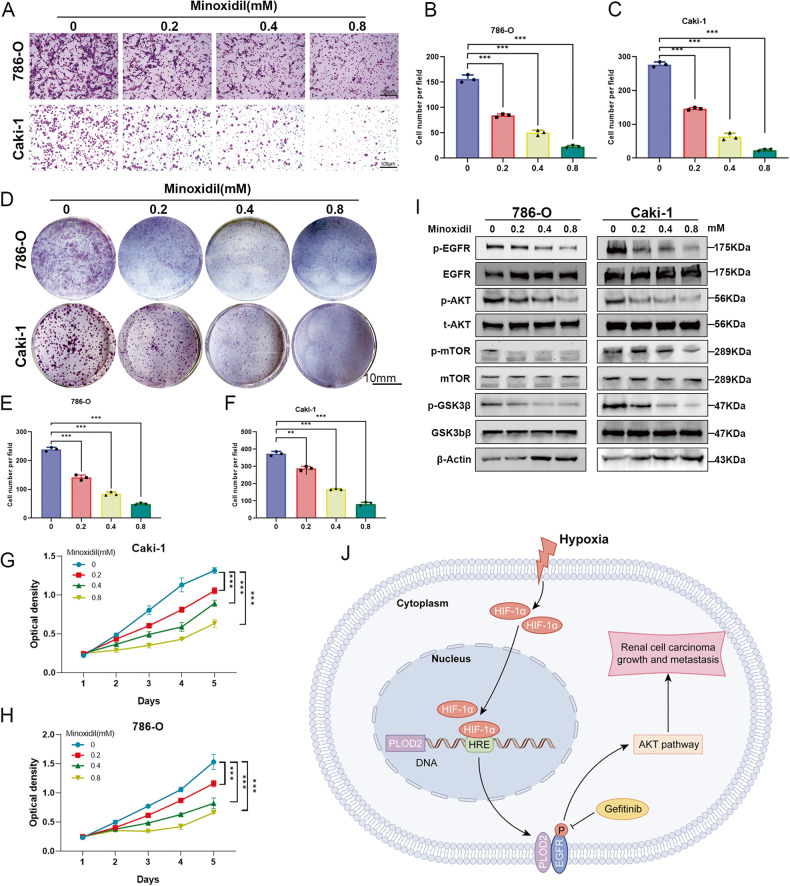
PLOD2 inhibitor MINOXIDIL attenuates ccRCC proliferation and migration via EGFR/AKT signaling axis. **A**–**C** Transwell assay and the statistical chart represent that PLOD2 inhibitor MINOXIDIL attenuates migration viability in a concentration-dependent manner. **D**–**F** The clone formation assay and the statistical chart represent that PLOD2 inhibitor MINOXIDIL attenuates clone formation proliferation in a concentration-dependent manner. **G**, **H** The MTT assay represents that PLOD2 inhibitor MINOXIDIL attenuates cell growth in a concentration-dependent manner. **I** Immunoblot results elucidate that MINOXIDIL attenuate EGFR, AKT, GSK3β, and mTOR phosphorylation level in a concentration-dependent manner. **J** A schematic model of HIF1A-PLOD2-EGFR/AKT signaling axis promotes clear cell renal cell carcinoma progression. Statistical significance was assessed using a two-tailed t-test. Error bars represent the standard deviations of three independent experiments. ****p < 0.001, **p < 0.01, *p < 0.05.

To investigate the practical application of PLOD2 inhibitors, we investigate the role of the novel PLOD2 inhibitor, Minoxidil [32], in the occurrence and progression of renal cancer. We conducted in vitro cultivation of 786-O and CAKI-1 cells to assess the effects of Minoxidil on cell migration and cell proliferation. The results indicate that Minoxidil significantly reduces the migration ability of both 786-O and Caki-1 cells (Fig. 8A–C). By examining the effects of Minoxidil on cell proliferation and clonogenicity in 786-O and CAKI-1 cells, we found that Minoxidil significantly reduces the proliferation activity and clone formation ability of both cell lines (Fig. 8D–H). Moreover, Minoxidil markedly reduces the expression of proteins including p-EGFR, p-AKT, p-GSK3β, and p-mTOR in both cell lines (Fig. 8I). These results indicate that Minoxidil can effectively suppress the progression of ccRCC, and this effect may be associated with the inactivation of the EGFR/AKT pathway.

## Discussion

The presence of hypoxia is a characteristic of many solid tumors, including clear cell renal cell carcinomas (ccRCC) [5]. HIF-1 is activated under low oxygen conditions, leading to the upregulation of downstream oncogenes. Existing literature has reported a certain correlation between PLOD2 and HIF1A in gastric cancer and endometrial carcinoma [33, 34]. However, the regulatory mechanism between them has not been elucidated in detail. In our study, we observed a significant positive correlation between PLOD2 and HIF1A in renal cancer tissues, with HIF1A promoting the expression of PLOD2.

Collagen is the most abundant protein in the human body and provides a structural foundation for the assembly of the extracellular matrix (ECM) [35]. It has been regarded as a “highway” for tumor cell migration and infiltration [36]. In the past, collagen was believed to act as a barrier that protects against cell migration and invasion [37]. However, recent research has shown that different collagen structures can influence cell migration [38]. PLOD2, an enzyme involved in collagen modification and ECM remodeling. It has been reported that PLOD2 exhibits significant upregulation in various cancers, and its elevation is closely associated with tumor progression [39, 40]. In our study, we found that PLOD2 expression was increased in ccRCC patients who had higher hypoxia level. Further investigation indicated that PLOD2 is directly regulated by HIF1A. Moreover, our clinical results confirmed that PLOD2 might contribute to ccRCC progression and occurrence. Our study revealed that PLOD2 could promote ccRCC cell proliferation, migration, tumor formation, and metastasis. In this study, Gene Set Enrichment Analysis (GSEA) revealed the regulatory role of PLOD2 in the EGF/EGFR signaling axis in ccRCC. EGFR, as a member of the ErbB transmembrane growth factor receptor family, plays a crucial biological role [41]. It can interact with various proteins expressed in different cell lines and influence the activity of other related proteins or enzymes by activating downstream genes [41]. EGFR is a complex network composed of extracellular ligand-binding domains, transmembrane hydrophobic regions, and intracellular tyrosine kinase activity regions, primarily involving transmembrane protein-ubiquitin subunits [42]. By binding to EGFR ligands, intracellular tyrosine kinases are activated, triggering the autophosphorylation process [43]. This mechanism allows cancer cells to generate various biological activities, making it a potential target for tumor therapy. The activation of EGFR has been widely confirmed to significantly promote cancer progression, metastasis, recurrence, and drug resistance [44, 45]. Our research findings demonstrate a direct interaction between PLOD2 and EGFR, leading to EGFR phosphorylation. A series of biochemical experiments indicate that PLOD2 interacts with the extracellular domains of EGFR, thereby affecting EGFR phosphorylation.

EGFR is a valuable target for treating several cancers, notably renal clear cell carcinoma [46]. However, it’s become increasingly resistant, and finding new inhibitors takes a long time. As mentioned above, PLOD2 has been confirmed as a potential therapeutic target for metastasis. The PLOD2 inhibitor Minoxidil has shown significant anti-metastatic effects. Therefore, PLOD2 might be a useful treatment target, even for patients resistant to EGFR inhibitors. Our findings open several avenues for future research. Firstly, the development of specific PLOD2 inhibitors and their evaluation in preclinical and clinical settings for ccRCC treatment should be a priority. Furthermore, investigating the potential synergistic effects of combining PLOD2 inhibitors with existing therapeutic approaches for ccRCC could hold promise.

Abnormal EGFR expression can regulate downstream oncogenic signaling pathways such as PI3K/AKT, Ras/Raf/MAPK, and JAK/STAT [47–49]. These pathways are closely associated with cancer proliferation, invasion, angiogenesis, and ultimately contribute to the malignant biological behavior of cancer [50–52]. Based on the analysis of the KEGG signaling pathways, it can be concluded that there is a close association between PLOD2 and the AKT pathway. Depletion of PLOD2 leads to decreased phosphorylation levels of AKT, GSK3β, and mTOR proteins, supported by immunoblotting. The role of the AKT-mTOR pathway in ccRCC has been extensively studied, and the downstream activation of the AKT-mTOR pathway may mediate the progression of ccRCC [53, 54]. This conclusion is further confirmed by establishing a cell culture model in this study. In summary, we have validated a novel signaling regulatory mechanism in the progression of ccRCC. In the hypoxic tumor microenvironment, the transcriptional regulation of HIF1A mediates the upregulation of PLOD2, which can activate EGFR phosphorylation, thereby activating the downstream AKT-mTOR pathway and ultimately promoting ccRCC tumor progression. This study indicated that PLOD2 may serve as a potential predictor and therapeutic target in ccRCC progression.

### Supplementary information

Supplementary legends

Figure S1

Figure S2

Table S1

Table S2

Reproducability checkist

Raw western blots

## Data Availability

Data will be made available on reasonable request.
