# Supplementary material for: Hypoxia-induced PLOD2 promotes clear cell renal cell carcinoma progression via modulating EGFR-dependent AKT pathway activation
Source: Cell Death Dis. 2023 Nov 27;14(11):774. doi: 10.1038/s41419-023-06298-7 (PMC10679098; doi:10.1038/s41419-023-06298-7)
Supplement: Supplementary file 1 — Supplementary legends [file 41419_2023_6298_MOESM1_ESM.docx]

**Figure S1. (A)** In the upper panel, a histogram shows the number of significantly differentially expressed genes for each cancer, and a heatmap shows fold change and FDR for each HRG. Significantly upregulated and downregulated genes have been highlighted in red and green, respectively. **(B)** Differences in HPIs between tumors and normal tissues among cancers based on TCGA pan-cancer RNA-seq data. **(C)** Determine the soft threshold power in WGCNA. Various soft-thresholding powers (β) and their scale-free fit indexes. **(D)** Using the 1-TOM dissimilarity measure, a dendrogram of gene clusters is shown. (E) The distribution of average gene significance and errors in the modules associated with hypoxia levels of ccRCC is shown. **(F)** Gene set enrichment analysis of the black module genes.

**Figure S2.** **(A)** This boxplot shows the expression of PLOD2 across tumor stages in TCGA-KIRC. **(B-C)** PLOD2 expression correlates with ccRCC stage in GSE53757 and GSE40435 cohorts. **(D)** Correlation between PLOD2 expression and Fuhrman grade for ccRCC based on microarray data from GSE40435. **(E)** Immunostaining images showing PLOD2 expression in ccRCC tissue microarrays at various stages and grades. The scale bar is 200mm. **(F)** The staining score for PLOD2 in ccRCC with low stage (I) and high stage (II-IV). **(G)** The staining score for PLOD2 in ccRCC with high grade (III/IV) and low grade(I/II). Statistical significance was assessed using a two-tailed t-test. ***: p < 0.001, *: p < 0.05.
