# Supplementary figures and images for: Hypoxia-induced PLOD2 promotes clear cell renal cell carcinoma progression via modulating EGFR-dependent AKT pathway activation
Source: Cell Death Dis. 2023 Nov 27;14(11):774. doi: 10.1038/s41419-023-06298-7 (PMC10679098; doi:10.1038/s41419-023-06298-7)

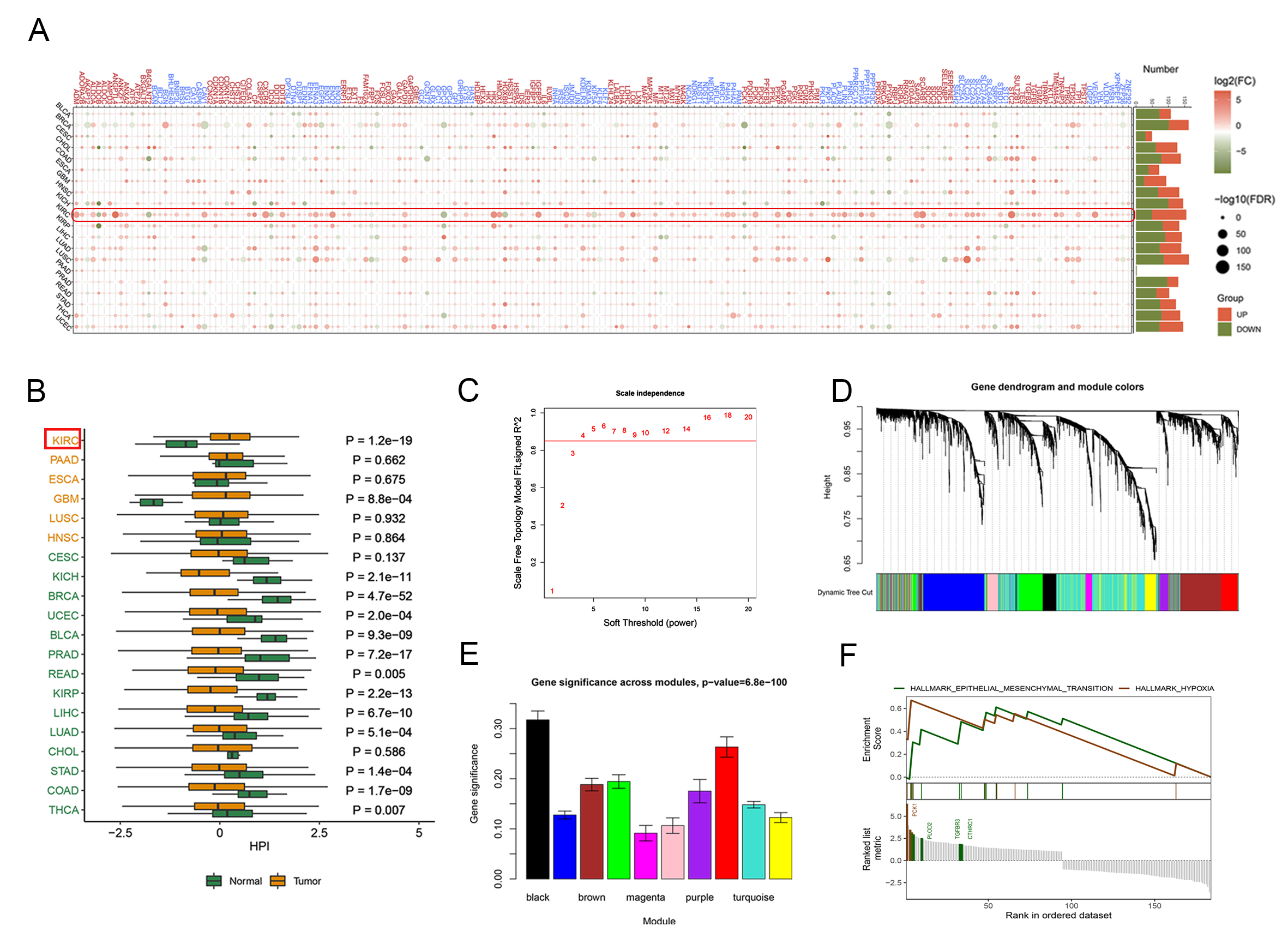

Supplement: Supplementary file 2 — Figure S1 [file 41419_2023_6298_MOESM2_ESM.tif]

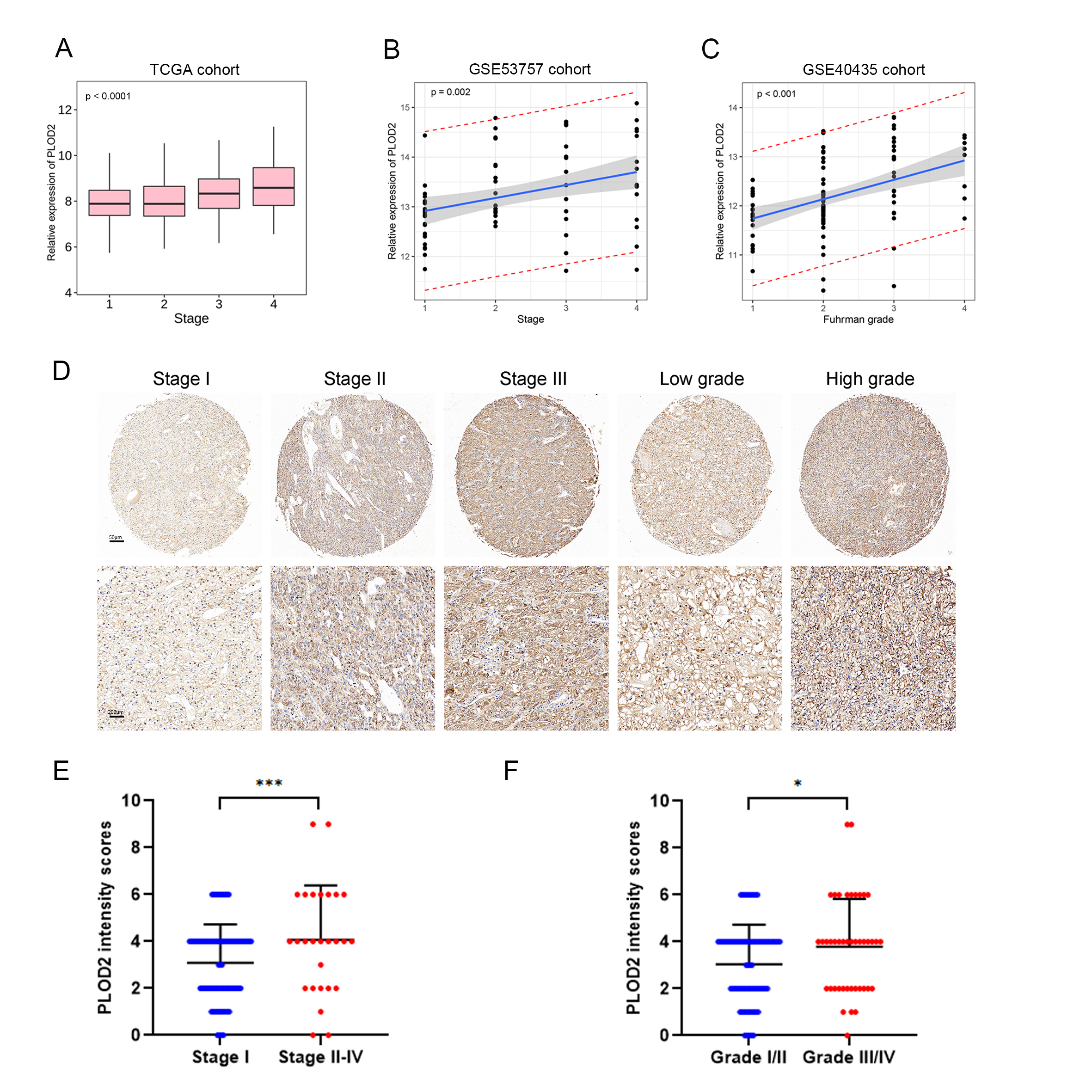

Supplement: Supplementary file 3 — Figure S2 [file 41419_2023_6298_MOESM3_ESM.tif]
