# Supplementary material for: Hypoxia-induced PLOD2 promotes clear cell renal cell carcinoma progression via modulating EGFR-dependent AKT pathway activation
Source: Cell Death Dis. 2023 Nov 27;14(11):774. doi: 10.1038/s41419-023-06298-7 (PMC10679098; doi:10.1038/s41419-023-06298-7)
Supplement: Supplementary file 4 — Table S1 [file 41419_2023_6298_MOESM4_ESM.docx]

**Table S1 The specific primer sequences**

| **ID** | **Primer** | Primer sequences **(5'to3')** | **Base count** |
| --- | --- | --- | --- |
| 1 | PLOD2-F | CATGGACACAGGATAATGGCTG | 22 |
| 2 | PLOD2-R | AGGGGTTGGTTGCTCAATAAAAA | 23 |
| 3 | HIF1A-F | GAACGTCGAAAAGAAAAGTCTCG | 23 |
| 4 | HIF1A-R | CCTTATCAAGATGCGAACTCACA | 23 |
| 5 | EGFR-F | AGGCACGAGTAACAAGCTCAC | 21 |
| 6 | EGFR-R | ATGAGGACATAACCAGCCACC | 21 |
| 7 | GAPDH-F | TGCACCACCAACTGCTTAG | 19 |
| 8 | GAPDH-R | GATGCAGGGATGATGTTC | 18 |
