# Supplementary material for: Hypoxia-induced PLOD2 promotes clear cell renal cell carcinoma progression via modulating EGFR-dependent AKT pathway activation
Source: Cell Death Dis. 2023 Nov 27;14(11):774. doi: 10.1038/s41419-023-06298-7 (PMC10679098; doi:10.1038/s41419-023-06298-7)
Supplement: Supplementary file 5 — Table S2 [file 41419_2023_6298_MOESM5_ESM.pdf]

Gene	mergedCol	GS.hist.g	MMblack	MMmagenta	MMturquoi	MMblue	MMpurple	MMbrown	MMred	MMgreen	MMpink
PCK1	black	-0.14753	-0.66963	-0.32372	-0.57991	0.314265	0.251142	-0.25651	0.461477	0.688565	0.230433
BDH2	black	-0.09747	-0.41909	-0.3724	-0.3872	0.126696	0.211975	-0.11245	0.351534	0.488649	0.302463
ANXA2	black	0.223198	0.696444	0.533571	0.421603	-0.6019	-0.03347	0.473632	-0.38449	-0.61515	-0.05119
TGFBI	black	0.520355	0.84507	0.376455	0.465142	-0.24905	-0.12309	0.179047	-0.56219	-0.58174	-0.17165
TIMP1	black	0.430928	0.790662	0.262589	0.455142	-0.23764	0.028491	0.270403	-0.50003	-0.63904	-0.18866
WDR72	black	-0.36221	-0.79706	-0.35755	-0.65818	0.261271	0.339186	-0.15422	0.585311	0.660042	0.273619
CEBPB	black	0.590167	0.761657	0.162518	0.431828	-0.36006	-0.17239	0.240562	-0.58267	-0.66696	-0.07335
IDH1	black	0.338703	0.709274	0.372275	0.435751	-0.5383	-0.10927	0.347128	-0.47598	-0.42781	0.100457
KCNJ15	black	-0.25944	-0.51415	-0.39707	-0.49135	0.266159	0.120779	-0.21619	0.453341	0.519455	0.167406
PLOD2	black	0.552222	0.794199	0.43051	0.381375	-0.24764	-0.32594	0.011448	-0.70088	-0.60065	-0.34256
ALDH3A2	black	-0.21127	-0.69603	-0.43906	-0.51187	0.486719	0.087723	-0.47887	0.370185	0.737925	0.062705
PERP	black	0.190205	0.477426	0.103286	0.121969	-0.1365	-0.17001	0.008521	-0.31983	-0.39366	-0.12776
AIF1L	black	-0.32375	-0.50524	-0.26127	-0.23918	-0.07653	0.179079	0.100457	0.546273	0.476494	0.43826
EFHD1	black	-0.33951	-0.66213	-0.41546	-0.4244	0.249019	0.476494	0.097466	0.675156	0.500997	0.248666
SHC1	black	0.491414	0.705672	0.197312	0.24307	-0.00248	-0.22625	-0.06811	-0.7041	-0.58923	-0.34018
MYOF	black	0.393048	0.688919	0.256737	0.247733	-0.24786	-0.23259	-0.00238	-0.58444	-0.46755	-0.18059
FLRT3	black	-0.46405	-0.50727	-0.22953	-0.24471	0.055824	0.173677	0.010965	0.55518	0.414818	0.312592
GPAT3	black	-0.30381	-0.81558	-0.49379	-0.61345	0.363174	0.211525	-0.21181	0.50328	0.575632	0.158242
ACTN1	black	0.369477	0.619718	0.102997	0.235803	-0.05534	0.144447	0.152068	-0.35195	-0.49418	-0.20831
CYS1	black	-0.52531	-0.79507	-0.35922	-0.42337	0.214162	0.126535	-0.09126	0.568718	0.507718	0.12123
AUH	black	-0.40363	-0.76828	-0.4581	-0.54283	0.198405	0.070455	-0.16384	0.541546	0.609107	0.236671
TMEM252	black	-0.17766	-0.5017	-0.32407	-0.41591	0.208759	0.072738	-0.22317	0.35028	0.478359	0.182166
C1R	black	0.438903	0.893627	0.415911	0.663065	-0.48932	-0.21786	0.305582	-0.58081	-0.65017	-0.0892
PTGDS	black	0.064056	0.334298	0.129108	0.093961	-0.32147	0.239662	0.371953	-0.06544	-0.2986	0.201074
MGAM	black	-0.0921	-0.49013	-0.35825	-0.48817	0.552962	0.098399	-0.51273	0.138851	0.51476	-0.00013
PCCA	black	-0.33816	-0.68908	-0.43958	-0.66969	0.226156	0.287253	-0.17564	0.560518	0.572288	0.291401
NPTX2	black	0.576436	0.383079	0.081066	0.000257	0.255065	-0.01383	-0.28774	-0.43893	-0.25915	-0.24159
NAMPT	black	0.576243	0.59917	0.121133	0.344524	-0.27979	-0.25047	0.134478	-0.52055	-0.58303	-0.15438
OSMR	black	0.587787	0.804007	0.228568	0.237186	-0.29211	-0.16059	0.079394	-0.64371	-0.57377	-0.17718
ALAD	black	-0.48852	-0.67072	-0.37806	-0.40607	0.147341	0.358351	-0.07515	0.602965	0.623641	0.302913
PDIA5	black	0.343688	0.332015	0.361888	0.100585	-0.09422	-0.102	-0.14374	-0.34176	-0.03126	-0.03685
ARHGAP24	black	-0.17107	-0.59731	-0.30989	-0.47222	0.369123	0.170429	-0.27909	0.374944	0.567689	0.140684
TGFBR3	black	-0.22947	-0.64158	-0.61567	-0.55785	0.488166	0.485369	-0.16065	0.436427	0.429545	0.068718
CTHRC1	black	0.513377	0.703421	0.203293	0.201138	-0.10782	-0.05399	0.072866	-0.50122	-0.55914	-0.25121

ARL4C	black	0.329957	0.697247	0.3042	0.5501	-0.28838	-0.17053	0.261464	-0.4654	-0.67779	-0.23445
FGG	black	0.207473	0.467297	0.433726	0.311017	-0.49482	-0.13419	0.345842	-0.33948	-0.35067	0.059103
FAM171A1	black	-0.42504	-0.70722	-0.38996	-0.3971	0.265355	0.332208	-0.05692	0.580262	0.552608	0.193549
TSPAN13	black	0.110554	0.164416	-0.14123	-0.09782	0.160782	0.104701	0.005885	-0.15827	-0.26915	-0.14686
AEBP1	black	0.432793	0.461766	-0.05634	0.040035	0.087691	0.246479	0.016657	-0.35391	-0.33407	-0.1393
NPNT	black	-0.5236	-0.68242	-0.25574	-0.51196	0.310374	0.493311	0.123674	0.696315	0.407968	0.152196
KMO	black	0.345103	0.639559	0.382565	0.365972	-0.39877	-0.02502	0.314039	-0.37192	-0.40504	-0.1066
CFB	black	0.371825	0.737732	0.426298	0.561612	-0.53852	-0.38552	0.162036	-0.51839	-0.41398	0.029294
FKBP11	black	0.27259	0.677954	0.425011	0.515081	-0.54586	-0.0365	0.391118	-0.35266	-0.39707	0.224194
BAG1	black	-0.17043	-0.56888	-0.49749	-0.50772	0.313654	0.209981	-0.17927	0.368802	0.399672	0.169979
PBX1	black	-0.47534	-0.62213	-0.19053	-0.38967	0.107885	0.384333	0.093125	0.598366	0.43051	0.177021
SPX	black	-0.18918	-0.39183	-0.30934	-0.32224	0.135186	-0.10075	-0.17213	0.22442	0.261142	0.156441
PKHD1	black	-0.39803	-0.73288	-0.43205	-0.54209	0.415268	0.118561	-0.34038	0.452634	0.58351	-0.03852
TGM2	black	0.409126	0.605087	0.152228	0.413467	-0.33224	-0.1786	0.195189	-0.43009	-0.40395	-0.01884
SPOCK1	black	0.315905	0.636954	0.220882	0.243617	-0.40787	-0.11026	0.298315	-0.31922	-0.50614	-0.07367
ZNRF3	black	-0.24191	-0.70818	-0.60978	-0.69246	0.407679	0.176314	-0.32517	0.40501	0.512123	0.129333
SOCS3	black	0.55518	0.452827	-0.02235	0.249373	0.002508	-0.16085	0.047431	-0.44212	-0.54843	-0.25622
FREM2	black	-0.35703	-0.78237	-0.39073	-0.51965	0.267059	0.17686	-0.26915	0.557367	0.778282	0.276899
CMC4	black	0.454595	0.358994	0.174384	-0.02939	0.084957	-0.246	-0.36015	-0.55232	-0.20181	-0.1057
NUCB2	black	0.228182	0.381729	0.296739	0.151939	-0.13091	-0.31385	-0.09817	-0.37578	-0.35932	-0.15384
SERPINE1	black	0.590167	0.563734	0.16538	0.341598	-0.01248	-0.04679	0.083639	-0.5127	-0.48556	-0.24555
MAP1B	black	0.29571	0.414496	0.086372	0.020098	-0.00389	0.353849	0.195029	-0.264	-0.4208	-0.18258
GRAMD1C	black	-0.316	-0.58206	-0.22776	-0.23313	0.093286	-0.11895	-0.21773	0.343527	0.598913	0.219918
PLAT	black	-0.43492	-0.65168	-0.42376	-0.58698	0.377163	0.531803	0.046112	0.590842	0.276513	-0.04817
DCDC2	black	-0.42414	-0.19667	0.098624	-0.02084	-0.07254	0.226928	0.299376	0.369767	0.152646	0.058364
TUBB6	black	0.4399	0.570615	0.196958	0.296546	-0.10316	-0.2158	0.051097	-0.48035	-0.45672	-0.03405
PTPN3	black	-0.44861	-0.79346	-0.34523	-0.44726	0.18998	0.04026	-0.13461	0.591774	0.606406	0.271304
EPB41L5	black	-0.46337	-0.67847	-0.33018	-0.53737	0.10647	0.32076	0.035115	0.635121	0.502605	0.21712
GPR155	black	-0.05261	-0.26738	-0.19914	0.058203	0.154351	0.07679	0.055373	0.249662	0.303589	0.130169
PLCL1	black	-0.32166	-0.66676	-0.46752	-0.63535	0.480288	0.572288	-0.04428	0.505306	0.332015	-0.04817
SSPN	black	0.391826	0.365072	0.147662	0.094797	0.004245	-0.11959	-0.23294	-0.34128	-0.21371	-0.15538
SLC2A1	black	0.508296	0.687665	0.274777	0.250884	-0.27153	-0.09004	0.113094	-0.51325	-0.44595	-0.07418
PLLP	black	-0.26844	-0.51897	-0.46331	-0.35697	0.143	-0.00244	-0.19831	0.423114	0.475047	0.240433
CDS1	black	-0.28819	-0.72246	-0.51872	-0.58013	0.115634	0.115281	-0.12908	0.536369	0.534118	0.312078
BICDL1	black	-0.46521	-0.45357	-0.02425	-0.04791	-0.31295	0.023281	0.261721	0.526786	0.432986	0.586694

IL17RD	black	-0.3296	-0.68358	-0.50968	-0.56788	0.540581	0.27304	-0.35919	0.444369	0.626053	-0.00624
MUC20	black	-0.23362	-0.51032	-0.17426	-0.2741	0.228182	0.171844	-0.07701	0.376037	0.494662	0.312207
CEACAM1	black	-0.24056	-0.62194	-0.25622	-0.46389	0.12615	0.267123	-0.15991	0.471863	0.631488	0.291723
SNHG12	black	0.082256	0.549039	0.395106	0.466815	-0.32513	-0.28407	0.17805	-0.43871	-0.4737	-0.12493
ITGA5	black	0.520741	0.643321	0.221558	0.313203	0.024246	-0.10268	0.03743	-0.55061	-0.58004	-0.25854
TBC1D13	black	-0.26015	-0.54859	-0.3032	-0.3225	0.101679	0.068429	-0.032	0.379092	0.492572	0.278603
ZNF385B	black	-0.42787	-0.33581	-0.06692	-0.13769	-0.11814	0.171329	0.157984	0.414657	0.18924	0.24664
ZNF710-AS	black	-0.22815	-0.66747	-0.2586	-0.28674	0.18924	0.107435	-0.14914	0.407518	0.557528	0.279182
SEMA5A	black	-0.35842	-0.61653	-0.42218	-0.63946	0.415043	0.543701	-0.08013	0.548685	0.501447	0.036626
NMB	black	0.06158	0.179529	-0.00939	-0.04885	0.219049	-0.10698	-0.26317	-0.29301	-0.15133	-0.23905
CA4	black	-0.17207	-0.69062	-0.56126	-0.6266	0.469323	0.455978	-0.21825	0.411248	0.48852	-0.05007
STMN3	black	0.108303	0.277542	0.156505	0.060358	-0.06502	-0.01724	-0.07843	-0.27989	-0.1867	-0.07772
TUB	black	-0.31021	-0.64049	-0.33037	-0.52363	0.418612	0.410123	-0.19966	0.47167	0.484372	-0.00058
SLC41A2	black	0.406071	0.397132	0.204322	0.162293	-0.29143	-0.34896	-0.27497	-0.44408	-0.00334	0.21757
TRHDE	black	-0.10136	-0.58624	-0.23648	-0.56978	0.349926	0.024503	-0.35375	0.250756	0.441057	-0.02116
DNASE1L3	black	-0.38607	-0.62059	-0.37443	-0.42427	0.357965	0.542993	0.111872	0.548267	0.283169	-0.01331
GRAMD4	black	0.21831	0.255	0.206669	0.087337	-0.05849	-0.35089	-0.24432	-0.37629	-0.2341	-0.08528
GPX8	black	0.239597	0.792109	0.582738	0.45556	-0.48862	-0.14258	0.346099	-0.44193	-0.61506	-0.11078
IRF6	black	-0.30851	-0.65133	-0.34449	-0.47961	0.119718	0.303524	0.03206	0.558493	0.504277	0.251656
C11orf71	black	-0.40668	-0.70503	-0.1958	-0.51035	0.123191	0.321725	-0.00563	0.713326	0.586276	0.255515
KIF2A	black	0.210946	0.781304	0.59827	0.58724	-0.58505	-0.17686	0.415332	-0.41125	-0.6028	-0.09676
MTURN	black	-0.46109	-0.7222	-0.42517	-0.47263	0.209692	0.498199	0.082513	0.630684	0.411666	0.102482
PDZRN3	black	-0.33812	-0.72796	-0.51544	-0.53621	0.435366	0.440253	-0.15972	0.522895	0.551418	0.026111
RUNX2	black	0.222587	0.646312	0.305936	0.36266	-0.18548	-0.09251	0.209338	-0.36195	-0.66168	-0.36504
YEATS2	black	0.372436	0.3552	0.142389	0.008714	0.20921	-0.06209	-0.20747	-0.33198	-0.1859	-0.38089
QSOX1	black	0.376937	0.684031	0.252942	0.389253	-0.44022	-0.23252	0.169529	-0.4471	-0.46489	0.037784
PTGFRN	black	0.360538	0.689916	0.438195	0.470416	-0.42575	-0.16303	0.293074	-0.38002	-0.4635	0.051772
RAB42	black	0.206508	0.618818	0.51923	0.480224	-0.37912	-0.39713	0.080327	-0.54	-0.44971	-0.08303
LBP	black	0.215737	0.430028	0.238858	0.279471	-0.29841	-0.03997	0.200527	-0.26362	-0.22828	0.053637
SLC43A3	black	0.406972	0.707248	0.407679	0.397003	-0.10817	-0.19406	0.025789	-0.54804	-0.62129	-0.32709
LINC01127	black	0.461091	0.649849	0.217281	0.210239	-0.20722	0.074346	0.164673	-0.32623	-0.47537	-0.10399
GXYLT2	black	0.505306	0.778185	0.267734	0.340665	-0.22381	-0.05943	0.120104	-0.51978	-0.58621	-0.20368
SLC2A11	black	-0.48855	-0.60965	-0.15155	-0.39353	0.093639	0.202103	0.002798	0.678532	0.548074	0.23143
MPPED2	black	-0.34227	-0.56142	-0.39006	-0.58393	0.465528	0.656505	0.063445	0.551032	0.23963	-0.04868
FAM13A-AS	black	0.351116	0.384398	0.141585	0.026079	0.183645	-0.27577	-0.32954	-0.5536	-0.32237	-0.40321

C10orf99	black	0.091453	0.500772	0.525532	0.298669	-0.43845	-0.17078	0.192006	-0.39585	-0.32243	0.091485
CENPV	black	-0.30082	-0.64798	-0.29458	-0.49524	0.299344	0.344524	-0.19654	0.432214	0.482121	0.097948
NICN1	black	-0.37199	-0.42543	-0.29298	-0.1056	0.102257	0.18297	0.21638	0.550711	0.293202	0.202553
MAP3K7CL	black	0.414882	0.635539	0.309312	0.30182	-0.16487	-0.02901	0.147791	-0.33536	-0.49096	-0.14364
SNHG15	black	0.385459	0.487041	0.203229	0.341598	-0.01733	-0.38327	-0.20667	-0.66615	-0.39089	-0.30803
SEMA3G	black	-0.2286	-0.50566	-0.3132	-0.46109	0.384366	0.542221	0.03997	0.492282	0.281079	-0.02219
PREX2	black	0.324844	0.423982	0.043765	0.113705	0.239983	-0.00177	-0.13416	-0.47788	-0.41511	-0.33832
APOB	black	0.26857	0.47704	0.434304	0.235932	-0.40951	-0.12718	0.12049	-0.3107	-0.1648	0.083028
PIP5K1B	black	-0.38121	-0.53081	-0.33745	-0.32539	0.281047	0.418998	0.049746	0.544697	0.274423	-0.01351
SEZ6L2	black	0.385716	0.512863	0.19863	0.209306	-0.16818	-0.49482	-0.17429	-0.52933	-0.34674	-0.02965
IL20RB	black	0.308026	0.658756	0.404335	0.397518	-0.39266	-0.2923	0.07203	-0.49961	-0.40745	-0.09695
FER1L4	black	0.278764	0.273008	0.008425	0.022477	0.137597	-0.22693	-0.25481	-0.37974	-0.16094	-0.25555
PHLDA3	black	0.305904	0.695575	0.360055	0.347418	-0.25458	-0.25831	0.002765	-0.54216	-0.50135	-0.17004
TRIM9	black	0.420316	0.446427	0.162197	0.012509	-0.02939	-0.16647	-0.19522	-0.49463	-0.33182	-0.32382
ASPHD1	black	0.276191	0.54846	0.347161	0.222683	-0.26905	-0.38527	0.013377	-0.48109	-0.42945	-0.06663
SLC6A18	black	-0.21432	-0.57013	-0.33613	-0.33099	0.352081	-0.05589	-0.31819	0.3224	0.479323	0.135764
ADA	black	0.421024	0.71577	0.420927	0.653354	-0.26487	-0.3279	0.147437	-0.6158	-0.62072	-0.20879
BTBD11	black	0.176217	0.648434	0.482025	0.456782	-0.59448	-0.14027	0.397968	-0.31462	-0.38884	0.080102
LHFPL3-AS	black	-0.44112	-0.68156	-0.3068	-0.35166	0.392726	0.063509	-0.22567	0.458872	0.450125	-0.08747
SPINK13	black	0.390829	0.638851	0.389414	0.271754	-0.30044	-0.17692	0.190141	-0.46611	-0.56843	-0.0892
TRHDE-AS1	black	-0.15168	-0.45791	-0.06833	-0.40057	0.177728	-0.03322	-0.12277	0.293331	0.306869	0.047849
SLC25A48	black	-0.34613	-0.72895	-0.38105	-0.39533	0.354492	0.138369	-0.29147	0.445141	0.639913	0.090231
AQP9	black	0.458615	0.66522	0.250145	0.277027	-0.46318	-0.21275	0.19445	-0.53717	-0.53566	0.01431
MYBL1	black	0.13734	0.601614	0.473632	0.421152	-0.28883	-0.1921	0.251592	-0.34378	-0.50222	-0.22484
EYA4	black	-0.40041	-0.63184	-0.4045	-0.61007	0.452955	0.519519	-0.11329	0.449257	0.365168	-0.09734
STUM	black	-0.29995	-0.66712	-0.37385	-0.50646	0.469966	0.247958	-0.24011	0.471284	0.513216	0.016046
ZNF684	black	-0.51962	-0.6913	-0.37121	-0.50039	0.207377	0.284777	-0.02235	0.593414	0.541771	0.161972
CYP4F12	black	0.038105	0.199498	0.17805	0.117692	-0.14554	0.022831	0.076114	-0.13991	-0.01614	0.033764
LOC101928	black	0.151392	0.667792	0.646376	0.491929	-0.49093	-0.18348	0.284198	-0.37285	-0.44173	-0.02019
MAMDC4	black	-0.23307	-0.41334	-0.14345	-0.15085	0.172358	-0.02527	-0.17773	0.255869	0.287285	0.066339
IL27RA	black	0.317255	0.609075	0.279632	0.372693	-0.32568	-0.31767	0.114509	-0.38083	-0.44492	-0.02447
MIR1915HG	black	-0.13982	-0.60033	-0.42112	-0.55865	0.458357	0.194546	-0.29333	0.269567	0.433115	-0.13766
ENAM	black	-0.37687	-0.70828	-0.28741	-0.37031	0.144865	0.136568	-0.06595	0.522767	0.453309	0.167953
LOC100506	black	0.352563	0.617307	0.486655	0.199241	-0.29462	-0.17348	0.060679	-0.54489	-0.4653	-0.15023
ATP8B3	black	0.395974	0.495948	0.252814	0.051772	-0.06229	-0.02971	-0.03328	-0.45913	-0.42848	-0.18037

ZNF582-DT	black	-0.34668	-0.55296	-0.37366	-0.45826	0.213904	0.464049	0.116856	0.463535	0.174802	-0.03975
ABCA17P	black	0.322207	0.478648	0.238536	0.176056	-0.14641	-0.24268	-0.01826	-0.47119	-0.28558	-0.16348
ZNF471	black	-0.28156	-0.63792	-0.47399	-0.49347	0.230465	0.322239	-0.10303	0.53206	0.441315	0.053155
CACNA2D4	black	0.294167	0.442247	0.438903	0.17924	-0.20857	-0.17432	-0.02656	-0.40138	-0.2859	-0.1449
CRABP1	black	0.096694	0.147566	-0.00068	-0.03602	-0.11335	0.196058	0.242717	0.012991	-0.08965	-0.0019
UNC5D	black	-0.35462	-0.4837	-0.13531	-0.20683	0.133964	0.140749	0.003087	0.4235	0.37414	0.096116
RERGL	black	-0.37009	-0.66152	-0.39003	-0.63007	0.413403	0.497492	-0.08296	0.547109	0.428677	0.047656
IGF2BP3	black	0.142324	0.625346	0.474854	0.332626	-0.49852	-0.13255	0.3816	-0.38504	-0.55586	0.0255
MUC3B	black	0.34462	0.520162	0.293106	0.345103	-0.40913	-0.21706	0.105955	-0.40823	-0.25831	0.156344
MYL3	black	-0.28249	-0.58142	-0.34066	-0.47042	0.317191	0.124124	-0.30124	0.246897	0.443051	0.017525
SCGB1D2	black	-0.33809	-0.62512	-0.19471	-0.45341	0.333365	0.196733	-0.32976	0.406972	0.574989	0.051193
AJAP1	black	-0.25265	-0.46984	-0.13216	-0.11522	0.195865	0.325326	0.174127	0.527044	0.232427	0.052897
PF4V1	black	0.257637	0.363657	0.214998	0.121969	-0.18824	-0.31243	-0.05817	-0.26947	-0.19075	0.024632
IQSEC3	black	-0.24677	-0.42572	-0.03409	-0.1247	0.088012	0.217409	0.000289	0.385266	0.427712	0.198502
FKBP10	black	0.367194	0.527783	0.20847	0.071162	-0.01489	-0.24124	-0.06779	-0.49884	-0.5473	-0.3234
DACT2	black	-0.37555	-0.44154	-0.15699	-0.13059	0.191556	0.141488	0.073317	0.361181	0.099749	-0.11789
LOC10272	black	-0.28606	-0.32404	0.150556	-0.04376	-0.00032	0.115763	0.167085	0.330471	0.253875	0.093254
LOC10099	black	-0.21423	-0.17593	6.43E-05	0.068461	-0.093	-0.04335	0.109975	0.169529	0.090392	0.044151
LOC65227	black	-0.28314	-0.2095	0.087337	-0.0656	-0.12248	0.041257	0.161972	0.282655	0.240691	0.30391
JPH2	black	0.330214	0.474307	0.225384	0.101421	-0.14782	0.157791	0.145411	-0.27375	-0.39999	-0.00151
NMU	black	0.223358	0.653032	0.414593	0.338929	-0.54171	-0.05277	0.436298	-0.38366	-0.60563	-0.07595
CCNYL2	black	0.323976	0.380475	0.238633	0.131134	0.111197	-0.23426	-0.20654	-0.45575	-0.28118	-0.40144
ATP6V1G2	black	-0.26298	-0.29352	-0.11695	-0.06669	0.143225	0.260981	0.099492	0.256544	0.202296	-0.14818
ADORA2B	black	0.390025	0.454852	0.300309	0.076468	-0.02836	-0.06492	0.08515	-0.28253	-0.4062	-0.16567
P4HA3	black	0.290919	0.51566	0.151521	0.179529	0.034922	-0.02968	-0.05872	-0.43771	-0.53364	-0.37411
RNF157-AS	black	-0.33128	-0.4135	-0.04148	-0.03515	-0.00534	0.171554	0.24217	0.374236	0.138433	0.08216
GYG2	black	0.146408	0.376037	0.292816	0.023185	-0.18664	0.182713	0.172005	-0.22175	-0.30526	-0.14062
TBX15	black	0.346003	0.422053	0.182713	-0.02338	-0.13985	-0.10171	0.046498	-0.32915	-0.40337	-0.13187
INHBE	black	0.37057	0.528362	0.380089	0.281497	-0.45205	-0.27989	0.077143	-0.29941	-0.28828	0.114059
C16orf74	black	0.251527	0.667438	0.447392	0.400283	-0.5738	-0.15872	0.250016	-0.3661	-0.33308	0.213679
LGALS12	black	0.28378	0.54489	0.443083	0.341533	-0.18853	-0.02749	0.099203	-0.35285	-0.36279	-0.1595
ABCA12	black	0.162486	0.492475	0.493794	0.150331	-0.30992	-0.28015	0.100553	-0.41893	-0.46508	-0.09219
CFAP20DC	black	0.205383	0.284841	0.206959	0.033443	-0.12425	-0.25002	-0.0692	-0.24069	-0.23133	-0.20567
BEST4	black	0.347418	0.359959	0.115023	0.055888	0.057914	-0.15133	-0.09936	-0.30574	-0.34041	-0.2578
CDRT15	black	-0.30938	-0.33105	-0.07216	-0.13223	0.127179	0.165091	0.070519	0.451508	0.291369	-0.05177

LINC0046C	black	0.059007	0.559586	0.351244	0.312914	-0.44263	-0.0301	0.35073	-0.22937	-0.51068	-0.02991
LOC101928	black	0.265644	0.297254	0.278056	0.08261	-0.10891	-0.21342	-0.03807	-0.33629	-0.30536	-0.10435
IGLON5	black	0.258795	0.411152	0.35655	0.047399	-0.28192	-0.04425	0.140556	-0.21924	-0.31272	-0.0675
TPRG1	black	0.336645	0.576982	0.524921	0.373465	-0.43652	-0.01257	0.293299	-0.34626	-0.45948	-0.10049
RHBDL2	black	0.166313	0.489581	0.442344	0.326902	-0.44778	-0.09988	0.348608	-0.2824	-0.48038	-0.13158
ADAMTS16-	black	-0.15914	-0.19471	-0.01592	0.002508	-0.06779	0.282623	0.313203	0.329603	0.214387	0.186475
PRG2	black	0.268828	0.267477	0.077047	0.045212	0.021095	-0.01978	-0.07148	-0.31864	-0.17676	-0.09493
MUC12	black	0.334137	0.476204	0.070005	0.277831	-0.25101	-0.2531	-0.02357	-0.48251	-0.32745	-0.03135
AICDA	black	0.169175	0.271657	0.187697	-0.0019	-0.1504	0.09573	0.057689	-0.07808	-0.15622	-0.01717
BTBD16	black	0.283684	0.432761	0.327995	0.082546	-0.22776	-0.08245	0.051161	-0.30642	-0.17471	-0.0511
