# Supplementary material for: Hypoxia-induced PLOD2 promotes clear cell renal cell carcinoma progression via modulating EGFR-dependent AKT pathway activation
Source: Cell Death Dis. 2023 Nov 27;14(11):774. doi: 10.1038/s41419-023-06298-7 (PMC10679098; doi:10.1038/s41419-023-06298-7)
Supplement: Supplementary file 7 — Raw western blots [file 41419_2023_6298_MOESM7_ESM.pdf]

Figure 2C

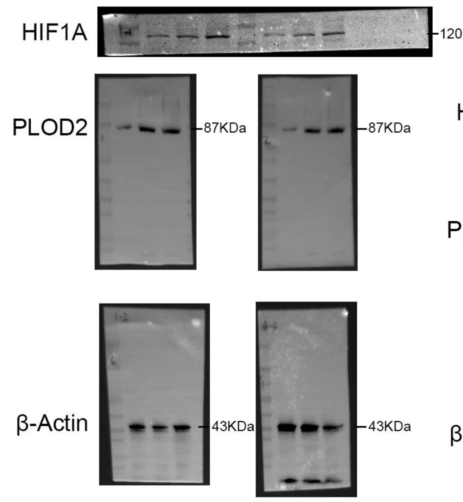

Figure 2H

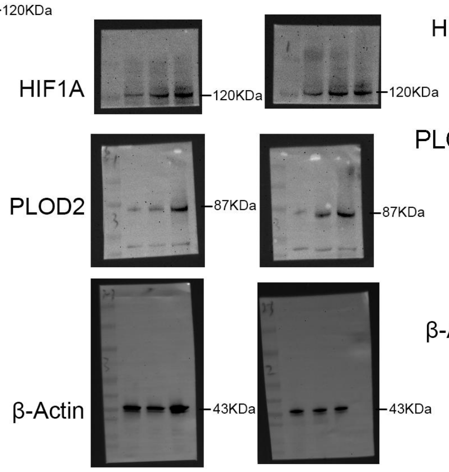

Figure 2I

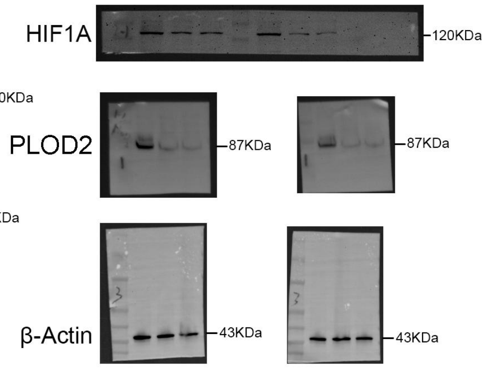

Figure 4B

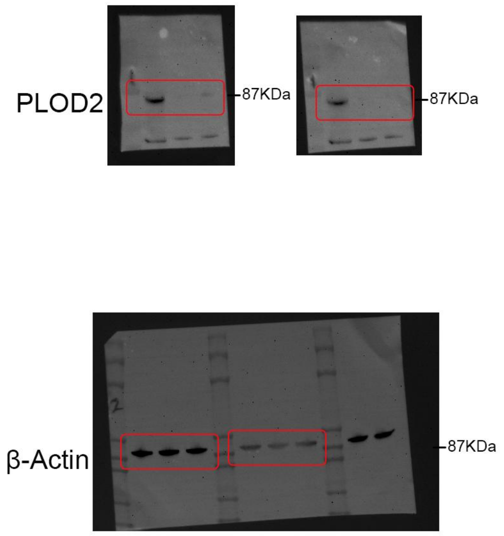

Figure 4L

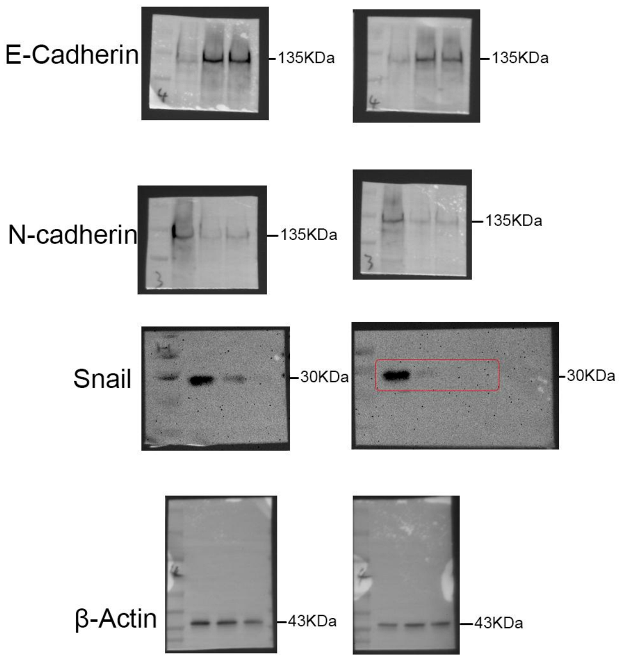

Figure 5A

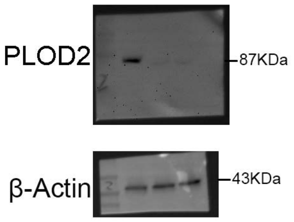

Figure 6B

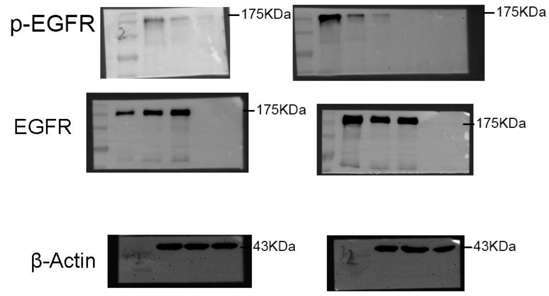

Figure 6C

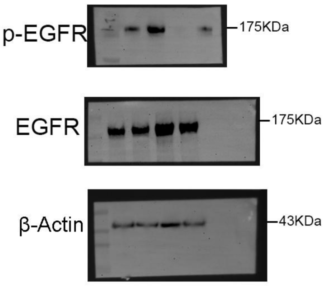

Figure 6D-E

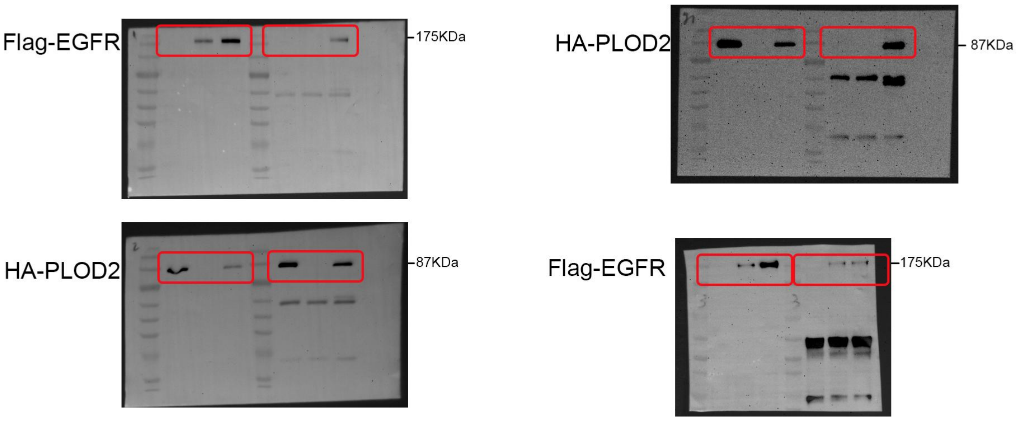

Figure 6G-H

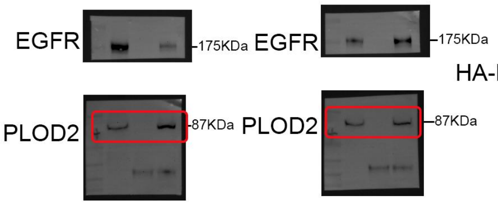

Figure 6J

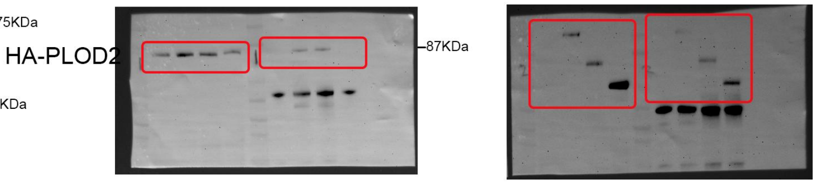

Figure 7B

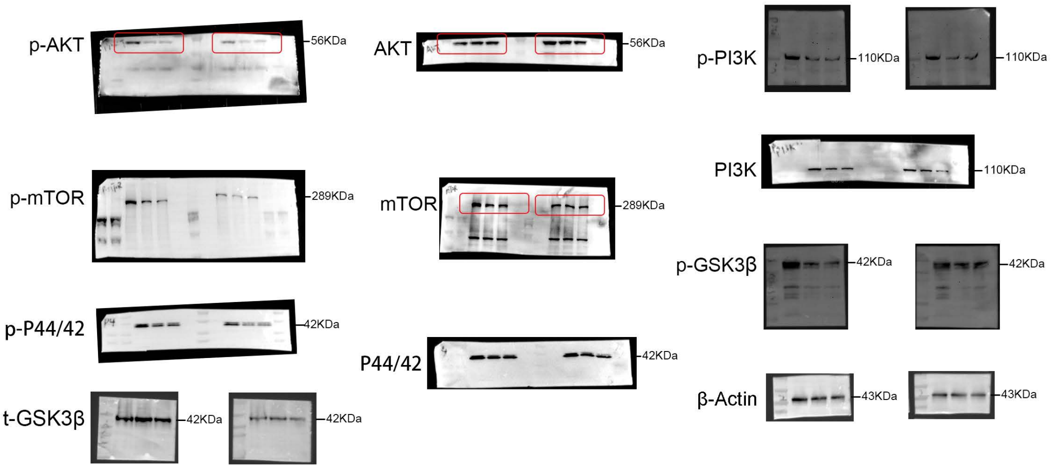

Figure 7C

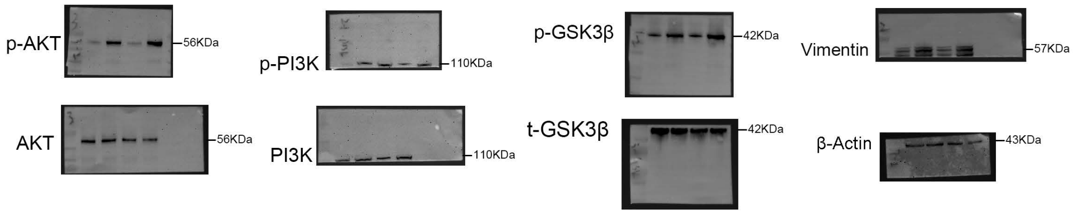

Figure 7L

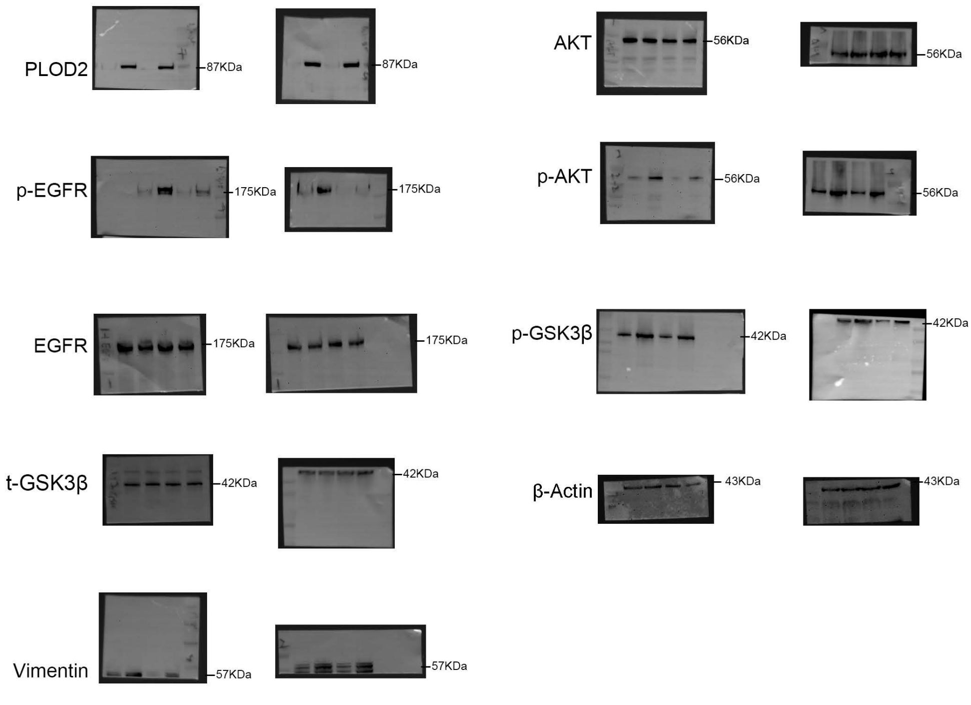

Figure 8L

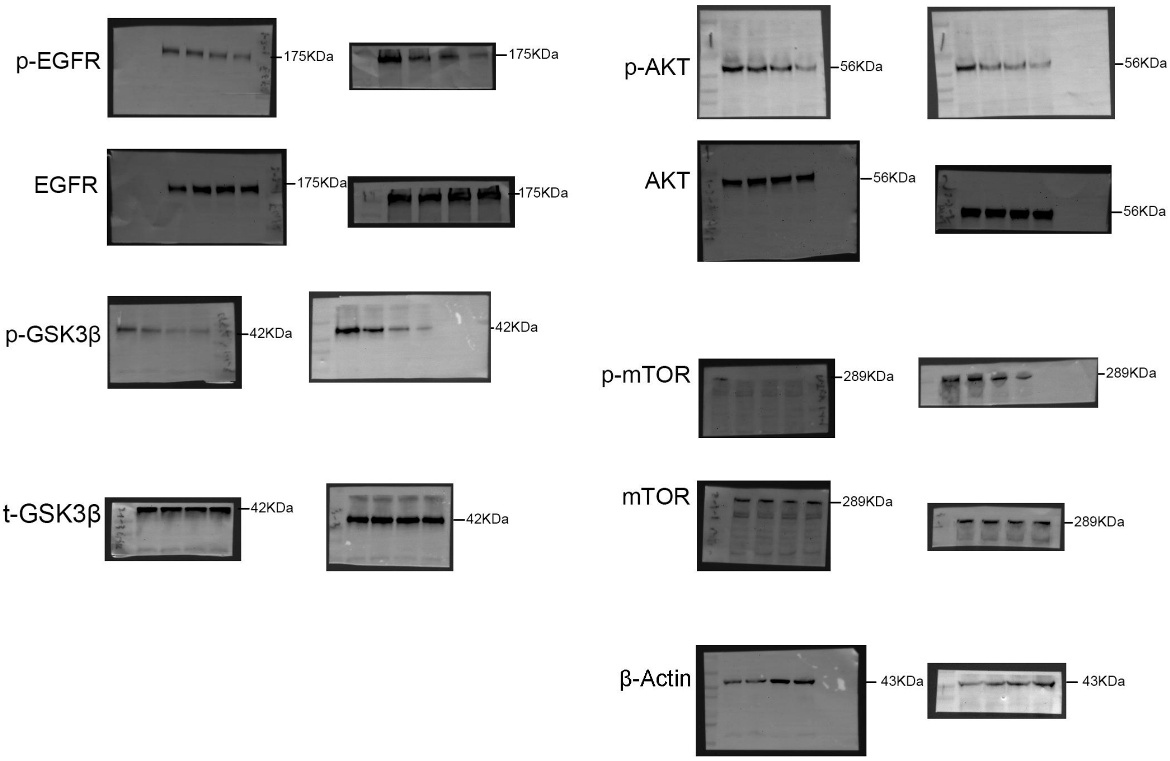
p-EGFR
175KDa
175KDa
EGFR
175KDa
175KDa
p-GSK3β
42KDa
42KDa
t-GSK3β
42KDa
42KDa
p-AKT
56KDa
56KDa
AKT
56KDa
56KDa
p-mTOR
289KDa
289KDa
mTOR
289KDa
289KDa
β-Actin
43KDa
43KDa
